# Berbamine hydrochloride as a brain-penetrant galectin-3 inhibitor in a model of Huntington’s disease

**DOI:** 10.1093/brain/awag069

**Published:** 2026-03-03

**Authors:** Jian Jing Siew, Chia-Wei Lee, Hui-Mei Chen, Hsien-Ya Lin, Yi-Ling Liu, Yi-Hsuan Lee, Ying-Ta Wu, Hung-Lin Chen, Fu-Tong Liu, Chun-Hung Lin, Yijuang Chern

**Affiliations:** Institute of Biomedical Sciences, Academia Sinica, Taipei 115, Taiwan; Institute of Biomedical Sciences, Academia Sinica, Taipei 115, Taiwan; Institute of Biomedical Sciences, Academia Sinica, Taipei 115, Taiwan; Institute of Biological Chemistry, Academia Sinica, Taipei 115, Taiwan; Genomics Research Center, Academia Sinica, Taipei 115, Taiwan; Department and Institute of Physiology, College of Medicine, National Yang Ming Chiao Tung University, Taipei 112, Taiwan; Brain Research Center, National Yang Ming Chiao Tung University, Taipei 112, Taiwan; Genomics Research Center, Academia Sinica, Taipei 115, Taiwan; Master Program in Clinical Genomics and Proteomics, Taipei Medical University, Taipei 110, Taiwan; Institute of Biomedical Sciences, Academia Sinica, Taipei 115, Taiwan; Department of Dermatology, Keck School of Medicine, University of Southern California, Los Angeles, CA 90033, USA; Institute of Biological Chemistry, Academia Sinica, Taipei 115, Taiwan; Institute of Biomedical Sciences, Academia Sinica, Taipei 115, Taiwan

**Keywords:** neurodegeneration, Huntington’s disease, microglia, galectin, drug screening

## Abstract

Galectin-3 (Gal3) acts as an intracellular sensor of lysosomal damage and a key driver of microglial activation and neuroinflammation in multiple neurodegenerative disorders. Despite its biological importance, no inhibitors have been identified that both modulate intracellular Gal3 activity and effectively cross the blood–brain barrier. Here, we established an innovative high-content imaging platform using microglia expressing Gal3-GFP, allowing direct visualization of Gal3 puncta formation upon lysosomal damage as a phenotypic readout for compound screening.

Through screening of ∼24 000 small molecules, we identified berbamine hydrochloride as a potent inhibitor of Gal3 puncta formation with confirmed blood–brain barrier permeability. Mechanistically, berbamine hydrochloride disrupts Gal3 oligomerization and Gal3–TREM2 interaction via a unique allosteric mechanism distinct from the canonical glycan-binding domain. It attenuated lipopolysaccharide-induced inflammation *in vitro* and achieved sustained brain exposure *in vivo*.

In the R6/2 mouse model of Huntington’s disease, berbamine hydrochloride effectively improved motor functions, reduced mutant huntingtin protein aggregation and restored crucial dopaminergic and ciliary signalling pathways. Transcriptomic profiling further identified Gal3 as a central regulatory hub within Huntington’s disease-associated networks that were corrected upon treatment. Computational docking and molecular dynamics simulations further supported an allosteric binding mode distinct from the canonical glycan-binding domain of Gal3.

Together, these findings establish berbamine hydrochloride as the first blood–brain barrier-penetrant small molecule inhibitor targeting intracellular Gal3 with therapeutic benefit in Huntington’s disease. Furthermore, the puncta-based screening strategy that we developed provides a robust platform for discovering intracellular modulators of Gal3 relevant to a wide range of neurological diseases.

## Teaser

Siew *et al.* identify berbamine hydrochloride as a brain-penetrant small molecule inhibitor of intracellular galectin-3, a protein that drives brain inflammation. Using a new cell-based screening method, they show that this compound reduces microglial activation and improves symptoms in a Huntington’s disease mouse model.

## Introduction

Galectin-3 (Gal3) is a member of the galectin family, characterized by its carbohydrate recognition domain (CRD), which mediates various cellular functions, including cell adhesion, proliferation, apoptosis, immune regulation and inflammation.^1^ Gal3 is expressed in multiple peripheral tissues, such as the gastrointestinal tract, heart, liver and kidney, whereas its basal expression in the CNS is relatively low in homeostatic conditions.^2^ However, Gal3 becomes markedly upregulated in response to injury, inflammation or neurodegeneration.^3^ Microglia are the predominant Gal3-expressing cells in the brain, where Gal3 regulates phagocytosis, lysosomal function and inflammatory signalling.^3^ Although astrocytes can express Gal3 in specific pathological contexts, microglial Gal3 is considered as the major contributor to disease-associated activity.^4^ Its involvement in neuroinflammation has been increasingly recognized, with studies indicating that Gal3 is upregulated in activated microglia, contributing to the inflammatory milieu associated with neurodegenerative conditions.^3^ Among these conditions, Gal3 has garnered significant attention owing to its multifaceted involvement in both physiological and pathological conditions. Earlier studies have shown that the level of Gal3 is low in young and healthy brains, but upregulated in various neurodegenerative diseases, brain injury and ageing, where it contributes to inflammation and neuronal dysfunction.^5,6^

Neurodegenerative diseases, such as Alzheimer’s disease (AD), Parkinson’s disease (PD) and Huntington’s disease (HD), are characterized by the accumulation of misfolded proteins, chronic neuroinflammation and the progressive loss of neuronal function. Specifically, HD is a genetic neurodegenerative disorder characterized by progressive motor dysfunction, cognitive decline and psychiatric symptoms.^7^ The disease-causing, mutant huntingtin protein forms aggregates in neurons, leading to neuronal dysfunction and cell death. Importantly, microglial activation plays a crucial role in the progression of HD, because these cells are responsible for the innate immune response in the brain.^8^ Gal3 has been implicated in the activation of microglia, the resident immune cells in the brain, thereby contributing to the sustained inflammatory response that exacerbates neurodegenerative conditions, including HD.^9^ Importantly, genetic deletion of Gal3 in mice has been demonstrated to be beneficial in multiple models of neurodegenerative diseases, including HD, AD and PD, suggesting that targeting Gal3 could be a viable therapeutic strategy.^3^

Despite its therapeutic potential, structure-based drug design for the full-length Gal3 protein remains challenging. Although the CRD of Gal3 is well characterized, facilitating the development of glycan-based inhibitors, such as TD139, knowledge about its N-terminal non-lectin domain that contains the intrinsically disordered region is still limited.^10^ Major gaps include understanding its dynamic conformational changes, oligomerization and interactions with intracellular components. These factors are crucial for designing inhibitors that go beyond targeting the extracellular glycan-binding activities of Gal3. Consequently, the development of small-molecule inhibitors capable of addressing the broader functional roles of Gal3, such as intracellular damage sensing,^1^ remains hindered by these knowledge gaps.

Another significant challenge remains in the development of effective Gal3 inhibitors capable of penetrating the blood–brain barrier (BBB).^11^ The BBB is a highly selective barrier that restricts the passage of substances from the blood into the CNS, thereby protecting the brain but also posing a major obstacle for drug delivery.^12^ Current pharmacological Gal3 inhibitors, such as TD139, exhibit limited efficacy in this regard, highlighting a crucial gap in the therapeutic landscape for neurodegenerative diseases. To address this challenge, small molecules with the ability to cross the BBB might offer a potential solution.^13^ For instance, the pharmacological inhibition of USP7 using a small molecule, eupalinolide B, significantly reduces microglial activation and neuronal injury in mouse models of senile dementia and PD.^14^

Recent studies have reported the presence of Gal3 puncta in the context of neurodegenerative diseases.^9,15^ Misfolded proteins in neurodegenerative diseases exhibit the ability to induce damage to intracellular vesicles, including endosomes, lysosomes and autophagosomes.^15^ This is of great interest because intracellular Gal3 acts as a damage sensor.^16^ Once Gal3 detects leakages in these vesicles, it translocates and accumulates at the damaged vesicles, by binding to the cytosolically exposed glycans present on the intraluminal membrane of the vesicles.^15,17^ By leveraging the ability of Gal3 to form puncta in response to intracellular vesicle damage, one can artificially induce vesicle damage and screen small molecule compounds that can inhibit Gal3 puncta formation as potential Gal3 inhibitors.

In this study, we used a novel, high-content screening approach, building on the newly recognized phenomenon of Gal3 puncta formation during lysosomal damage to identify and validate potential inhibitors of Gal3. This primary screen was complemented by a homogeneous time-resolved fluorescence (HTRF)-based assay to validate the inhibition of Gal3 interaction with the glycoprotein TREM2 (triggering receptor expressed on myeloid cells 2), in addition to Gal3 self-association, and to strengthen hit identification. Among the 23 936 compounds screened, we identified berbamine hydrochloride as a promising candidate that not only inhibits Gal3 but also exhibits BBB permeability. Beyond Gal3 inhibition, berbamine hydrochloride reduced microglial activation, improved neuronal markers, reduced the abnormal activation of astrocytes, modulated Gal3-associated transcriptional networks and ameliorated motor deficits in R6/2 HD mice. The identification of berbamine hydrochloride as a Gal3 inhibitor is particularly noteworthy because it represents a crucial strategy to mitigate the detrimental effects of Gal3 in the context of neurodegeneration. Our findings highlight the effectiveness of our Gal3 puncta-based high-content screening approach in identifying new therapeutics for complex neurological disorders and provide a promising lead for future investigations into Gal3-targeted therapies.

## Materials and methods

Materials and methods are provided in the Supplementary material.

## Results

### Identification and validation of Gal3 inhibitors using high-content screening

To identify potential Gal3 inhibitors, we established a stable mouse microglial cell line (SM826^18^) expressing Gal3 fused with green fluorescent protein (Gal3-GFP) and a control line expressing only GFP. Western blot analysis confirmed the stable expression of Gal3-GFP and endogenous Gal3 in SM826 stable cells (SM826-Gal3-GFP) (Fig. 1A). The screening strategy involved inducing lysosomal damage with L-leucyl-L-leucine methyl ester (LLOMe), which allows Gal3-GFP to accumulate at the damaged lysosomes by binding to the glycans residing in the lysosomes and forming puncta. Compounds that effectively reduced the formation of Gal3 puncta in the presence of LLOMe were considered potential Gal3 inhibitors (Fig. 1B). After treatment with LLOMe for 2 h, SM826-Gal3-GFP cells exhibited a significant increase in Gal3 puncta compared with the dimethyl sulphoxide-treated control (Fig. 1C and D and Supplementary Fig. 1A). In this high-throughput screen, compounds that suppressed Gal3 puncta formation following LLOMe treatment were defined as positive hits, whereas compounds that failed to inhibit Gal3 puncta formation were classified as non-hits. Notably, control cells expressing GFP alone did not form puncta when treated with LLOMe (Fig. 1D).

**Figure 1:**
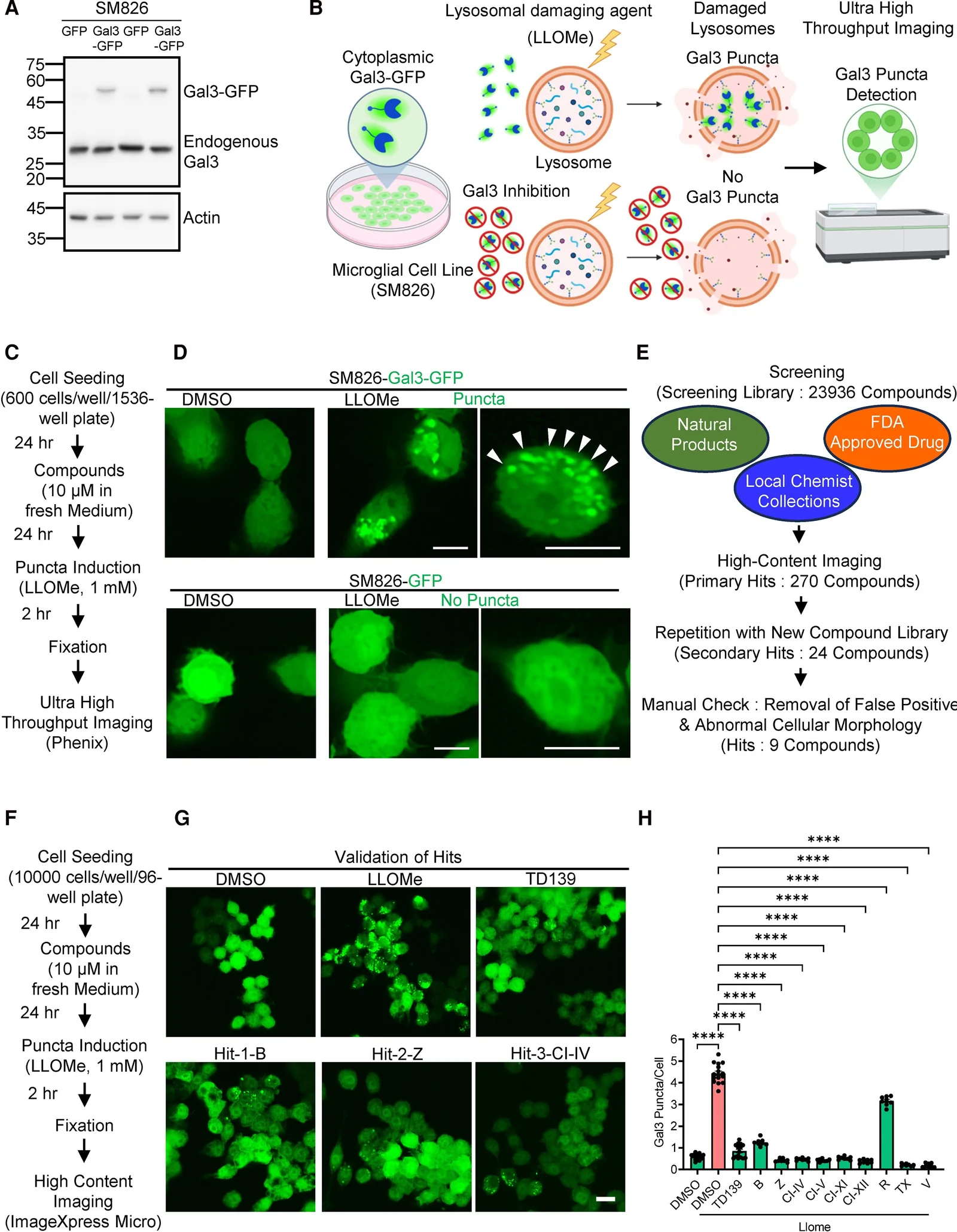
**Identification and validation of galectin-3 inhibitors using high-content screening in a microglial cell line.** (**A**) Western blot analysis of SM826 microglial cell line stably expressing Gal3-GFP or GFP control, showing expression levels of Gal3 fused with GFP, endogenous Gal3, and actin as the loading control. (**B**) Schematic illustration of Gal3 inhibitor screening. Microglial cells expressing Gal3-GFP were treated with a lysosomal damaging agent (LLOMe) to induce Gal3 puncta formation. Cells showing a reduction in Gal3 puncta indicate successful inhibition. Detection was performed using ultra-high throughput imaging. Created in BioRender. Chang, M. (2026) https://BioRender.com/x2bd3vg. (**C**) Workflow for ultra-high-throughput screening in a 1536-well plate format. (**D**) Representative images showing Gal3-GFP puncta formation in SM826 cells treated with LLOMe compared with the absence of puncta in cells treated with DMSO and cells expressing GFP alone. (**E**) Overview of the screening approach, including initial compound library screening (23 936 compounds), followed by high-content imaging to identify primary and secondary hits, with manual verification of final hits. (**F**) Workflow for validation of hit compounds in a 96-well plate format. (**G**) Representative images of Gal3 puncta in cells treated with LLOMe, DMSO, TD139 (positive control) and selected hit compounds (Hit-1-B, Hit-2-Z and Hit-3-Cl-IV). (**H**) Quantification of Gal3 puncta per cell in different treatment groups; *n* = 15 for DMSO and LLOMe + TD139, *n* = 16 for DMSO + LLOMe, and *n* = 8 for other compounds. Scale bars = 10 µm. Data were analysed by one-way ANOVA with Tukey’s test and are presented as the mean ± standard error of the mean; ****P < 0.0001. B = berbamine hydrochloride; CI-IV = calpain inhibitor IV; CI-V = calpain inhibitor V; CI-XI = calpain inhibitor XI; CI-XII = (RAC)-calpain inhibitor XII; DMSO = dimethyl sulphoxide; Gal3 = galectin-3; GFP = green fluorescent protein; R = ryuvidine; TX = TX-1123; V = valtrate; Z = Z-FA-FMK.

Our initial ultra-high-throughput screening, which involved 23 936 compounds in a 1536-well plate format, resulted in the identification of 270 primary hits. Subsequent secondary screening, using these compounds retrieved from a freshly prepared compound library and testing them in a dose-dependent manner, narrowed the hits down further, to 24. Manual examination of these 24 compounds led to the exclusion of false positives, in addition to treatment-induced abnormal cellular morphology, indicating potential toxicity (Supplementary Fig. 1B–D). Ultimately, nine promising candidates that successfully inhibited Gal3 puncta formation were selected for further characterization (Fig. 1C–E). These hits were then validated using different batches of compounds in a 96-well plate format for consistency (Fig. 1F and G). Quantification of Gal3 puncta per cell demonstrated significant reductions in puncta upon treatment with berbamine hydrochloride, Z-FA-FMK, four calpain inhibitors, ryuvidine, TX-1123 or valtrate (Fig. 1H), indicating that they are potent inhibitors of Gal3.

### Gal3 binding inhibition and functional assays

To assess whether these identified compounds directly inhibit Gal3, we next performed an HTRF assay to determine whether they interfered with the interaction between Gal3 and TREM2, in addition to Gal3 self-association. TREM2 is a glycoprotein and microglial surface receptor involved in lipid sensing and innate immune signalling,^19^ and Gal3 is known to bind to TREM2 through its CRD in a carbohydrate-dependent manner.^4^ This cell-free biochemical assay was used as a secondary validation step to distinguish direct Gal3 binders from compounds that indirectly reduce Gal3 puncta in the primary cell-based screen. The HTRF assay showed that berbamine hydrochloride, Z-FA-FMK, calpain inhibitors IV and V, (RAC)-calpain inhibitor XII, TX-1123 and valtrate all inhibited the Gal3–TREM2 binding by >10% (Fig. 2A). In particular, berbamine hydrochloride and Z-FA-FMK inhibited Gal3–TREM2 binding by 59.37% and 16.94%, respectively. We also tested the effect of the compounds on the self-association property of Gal3, which is mediated by its N-terminal domain,.^20,21^ Only berbamine hydrochloride and Z-FA-FMK inhibited Gal3 self-association by >10%, with inhibition levels of 56.49% and 26.10%, respectively (Fig. 2B). These results positioned berbamine hydrochloride and Z-FA-FMK as potent inhibitors of both Gal3–TREM2 interaction and Gal3 self-association, comparable to a well-known Gal3 inhibitor (TD139^22^) (Fig. 2A and B). Owing to solubility issues in the HTRF buffer, ryuvidine was excluded from this assay. We thus selected berbamine hydrochloride and Z-FA-FMK for further analysis.

**Figure 2:**
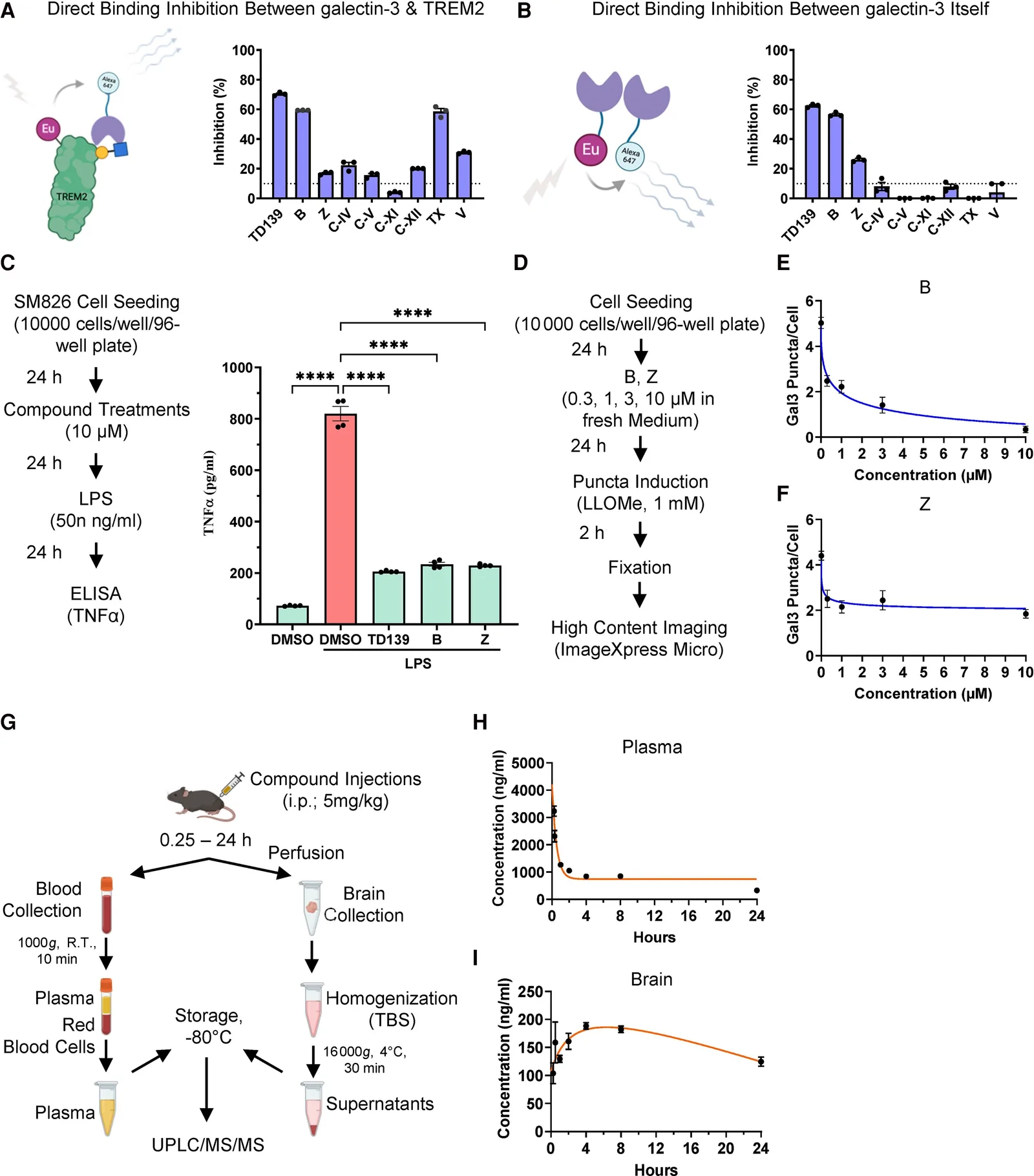
**Evaluation of Gal3 binding inhibition and compounds that can cross the blood–brain barrier.** (**A**) Direct binding inhibition between Gal3 and TREM2 measured using the homogeneous time-resolved fluorescence (HTRF) assay. The binding between Gal3 and TREM2 is carbohydrate recognition domain (CRD) and carbohydrate dependent. Hit compounds were assessed for their ability to inhibit the Gal3–TREM2 interaction, with TD139 serving as the positive control, *n* = 3. Data are presented as the mean ± standard error of the mean (SEM). (**B**) Direct binding inhibition between Gal3 itself, also measured using the HTRF assay. The binding between Gal3 is N-terminal domain (NTD) dependent. Hit compounds were evaluated for their ability to disrupt Gal3 self-association, *n* = 3. Data are presented as the mean ± SEM. (**C**) Inflammatory response inhibition in SM826 cells treated with hit compounds (berbamine hydrochloride and Z-FA-FMK) and stimulated with LPS. TNFα levels were measured by ELISA, *n* = 4. Data were analysed by one-way ANOVA with Tukey’s test and are presented as the mean ± SEM; ****P < 0.0001. (**D**) Workflow for dose-dependent study for berbamine hydrochloride and Z-FA-FMK in a 96-well plate format. (**E** and **F**) Quantification of Gal3 puncta per cell in response to increasing doses of berbamine hydrochloride (**E**) and Z-FA-FMK (**F**). Data are presented as the mean ± SEM. (**G**) Workflow for the blood–brain barrier permeability assay in mice. Compounds (5 mg/kg) were injected intraperitoneally, followed by blood and brain collection at specified time points. Plasma and brain tissue homogenates were analysed by ultra-performance liquid chromatography/tandem mass spectrometry (UPLC/MS/MS). Created in BioRender. Chang, M. (2026) https://BioRender.com/osa5mc8. (**H** and **I**) Plasma and brain concentrations of the hit compound, berbamine hydrochloride, over time (15 min, 30 min, 1, 2, 4, 8 and 24 h), *n* = 4. Each dot represents an individual measurement at a given time point, starting from 15 min. Data are presented as the mean ± SEM. Orange line indicates a model fitted using non-linear regression analysis. B = berbamine hydrochloride; CI-IV = calpain inhibitor IV; CI-V = calpain inhibitor V; CI-XI = calpain inhibitor XI; CI-XII = (RAC)-calpain inhibitor XII; Gal3 = galectin-3; LPS = lipopolysaccharide; TX = TX-1123; V = valtrate; Z = Z-FA-FMK.

Given that inhibition of Gal3 has previously been shown to suppress the pro-inflammatory response and impair toll-like receptor 4 signalling,^9,23^ we evaluated the inflammatory response in SM826 microglial cells exposed to lipopolysaccharide (LPS) and treated with the indicated hit compound (Fig. 2C). ELISA revealed that berbamine hydrochloride and Z-FA-FMK significantly reduced the LPS-induced secretion of tumour necrosis factor alpha (TNFα), with TD139 serving as the positive control. Using the cell-based assay (Fig. 2D–F), the IC_50_ values of berbamine hydrochloride and Z-FA-FMK for reducing Gal3 puncta formation in the conditions tested were 0.98 and 0.05 μM, respectively.

### Assessment of blood–brain barrier permeability

We next investigated the ability of berbamine hydrochloride and Z-FA-FMK to cross the BBB (Fig. 2G). Mice were injected intraperitoneally with 5 mg/kg of berbamine hydrochloride or Z-FA-FMK. Blood and brain tissues were collected at several time points post-injection (15 min, 30 min, 1, 2, 4, 8 and 24 h). Ultra-performance liquid chromatography/tandem mass spectrometry (UPLC/MS/MS) analysis detected both berbamine hydrochloride (Fig. 2G–I) and Z-FA-FMK (Supplementary Fig. 2) in the brain, demonstrating their ability to cross the BBB. This property is crucial, because the BBB represents a major obstacle in the delivery of therapeutic agents to the CNS. Berbamine hydrochloride is of great interest because, although it exhibits rapid clearance from plasma within the first 1–2 h (Fig. 2H), its pharmacokinetic profile reveals favourable brain exposure (Fig. 2I). Approximately 0.05%–0.09% of the injected dose was detected in the brain across the time course. Notably, brain concentrations of berbamine hydrochloride increased over time, rising from 104 ng/ml at 15 min to 159 ng/ml at 30 min, 130 ng/ml at 1 h, and 161 ng/ml at 2 h. Concentrations reached a sustained peak of 189 ng/ml at 4 h and remained high, at 182 ng/ml, at 8 h. Importantly, a measurable level of 125 ng/ml was still detected at 24 h post-injection. This delayed accumulation and prolonged retention suggest effective penetration across the BBB and the potential for sustained target engagement within the CNS, highlighting the possibility for neurological applications. Conversely, Z-FA-FMK exhibited a rapid clearance from both plasma and brain shortly after administration (Supplementary Fig. 2). This lack of sustained presence in the brain suggests limited potential for effective target engagement and therapeutic efficacy, leading us to exclude it from further investigation.

### Docking and molecular dynamics simulations of berbamine binding to Gal3

To predict the binding of berbamine hydrochloride, we performed docking simulations of berbamine with full-length Gal3 and its CRD using AutoDock Vina.^24^ The docking results suggested that berbamine binds to the F-face near β7, β8 and β9 in both cases (Supplementary Fig. 3A and B), which is not the carbohydrate-binding site of Gal3. This finding is consistent with results from nuclear magnetic resonance experiments showing the binding between PTX-008 and Gal3. PTX-008 is an angiostatic agent that binds to the non-CRD regions of Gal3, induces conformational changes and affects its CRD functions.^25^ Notably, in the full-length protein, the N-terminal domain (NTD), residues 1–50, appears to contribute to the binding, with W22 and W26 being particularly significant.^25^ Additionally, we performed docking simulation of Gal3 with TD139 and compared the docking results with the crystal structure of Gal3 CRD bound to TD139 as a control (Supplementary Fig. 4). From the crystal structure in Protein Data Bank (PDB code: 5h9p), TD139 binds to Gal3 CRD at a pocket containing W181 (Supplementary Fig. 4A), which is consistent with docking results of the full-length Gal3 protein and CRD (Supplementary Fig. 4B and C), indicating the validity of docking set-up.

To investigate the stability of berbamine binding further, we performed 10 ns molecular dynamics simulations. In the full-length Gal3 protein, berbamine remained stably bound to the F-face throughout the simulation. However, when berbamine was made to bind to the CRD, the compound dissociated from the predicted F-face binding site at 4 ns. Additionally, we tested whether berbamine could bind stably to the S-face of the CRD. The results showed that the compound left the binding pocket within 2 ns. These findings suggest that the NTD might play an important role in stabilizing berbamine binding to the F-face and indicate that its binding mode might resemble that of PTX-008.

### Therapeutic effects of berbamine hydrochloride in an HD mouse model (R6/2)

HD is characterized by the progressive loss of motor coordination and body weight, accompanied by neuroinflammation and neuronal dysfunction.^7^ Neuroinflammation, driven by activated microglia, plays a significant role in HD progression.^9,26^ To evaluate the therapeutic potential of berbamine hydrochloride, its effects were tested in an HD mouse model (R6/2).^27^ The R6/2 line carries an expanded CAG repeat in the human HTT exon 1 transgene and develops progressive motor deficits, body weight loss and neuroinflammation. Because of its rapid and robust disease phenotype, the R6/2 model is widely used for preclinical therapeutic testing. We have previously demonstrated that genetic suppression of Gal3 in R6/2 mice ameliorates neuroinflammatory responses and improves motor outcomes, supporting both the role of Gal3 in HD pathogenesis and the suitability of the R6/2 model for evaluating Gal3-targeted interventions.^9^ Mice were injected intraperitoneally with berbamine hydrochloride (5 mg/kg, 5 days per week), starting at the age of 6 weeks, and were sacrificed at 12.5 weeks (Fig. 3A). Behavioural assessments demonstrated that berbamine hydrochloride significantly delayed the impairment of motor function, as assessed by the rotarod performance test (Fig. 3B), and the progression of neurological deficits, evaluated by clasping behaviour (Fig. 3C). No specific effect was observed on body weight loss, a common feature of HD mice (Fig. 3D).

**Figure 3:**
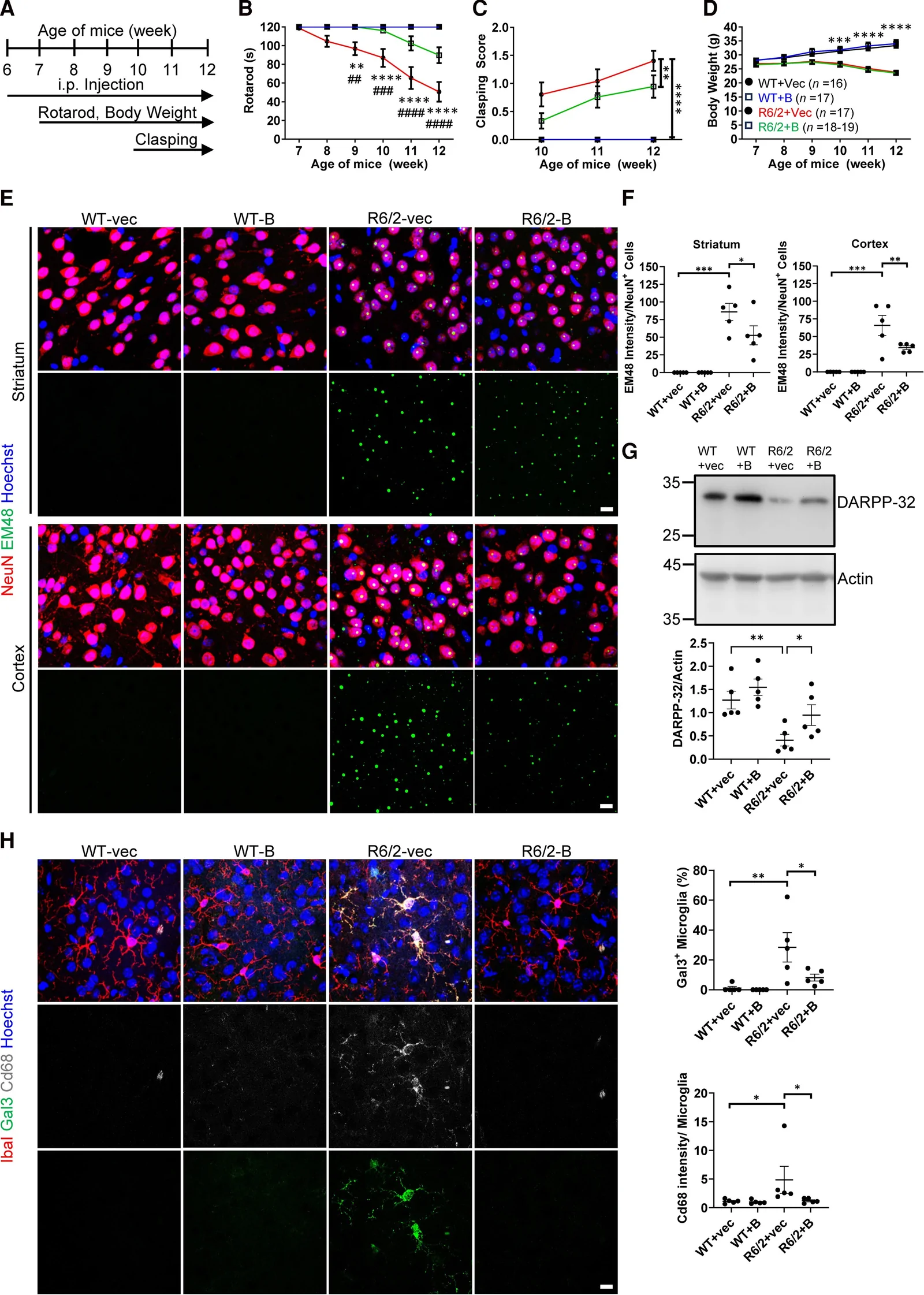
**Evaluation of behavioural, histological and molecular changes in Huntington’s disease mice treated with berbamine hydrochloride.** (**A**) Time line for intraperitoneal injections, behavioural testing and body weight measurements in HD mice (R6/2) and their littermate controls (WT). (**B**) Rotarod performance across different treatment groups over time, WT and R6/2 mice were treated with vehicle (vec) or berbamine hydrochloride (**B**). (**C**) Clasping scores of mice at the indicated ages. Data represent the mean ± standard error of the mean (SEM); asterisks indicate specific comparisons between WT and R6/2 mice treated with vehicle, and hashtag symbols indicate specific comparisons between R6/2 mice treated with B and vehicle. (**D**) Body weight measurements of mice during the experimental time line, *n* = 15 for WT + vec, *n* = 17 for WT + B and R6/2 + vec, *n* = 19 for R6/2 + B. (**E**) Immunofluorescence staining of NeuN (red) and EM48 (green) in the striatum and cortex of treated and control mice. Hoechst staining (blue) was used to label nuclei. Scale bars = 10 µm. (**F**) Quantification of EM48 intensity per NeuN-positive cell in the striatum and cortex, showing differences between treatment groups, *n* = 5. (**G**) Western blot analysis of DARPP-32 protein expression in the striatum, with actin as the loading control, *n* = 5. (**H**) Immunofluorescence staining of the microglia marker Iba1 (red), Gal3 (green) and Cd68 (grey) in the striatum of treated and control mice, showing microglial activation. Hoechst staining (blue) was used to label nuclei, *n* = 5. Data are presented as the mean ± SEM and analysed using two-way ANOVA with Tukey’s test; *P < 0.05, **P < 0.01, ***P < 0.001 and ****P < 0.0001. Scale bars = 10 µm. B = berbamine hydrochloride; HD = Huntington’s disease; WT = wild-type.

Histological analysis revealed that berbamine hydrochloride decreased the aggregation of mutant Huntingtin (i.e. EM48-positive) in the striatum and cortex compared with the vehicle-treated controls (Fig. 3E and F). Reductions in EM48 immunoreactivity reflect an overall decrease in mutant huntingtin accumulation, probably attributable to combined effects on aggregate number and size. Previous studies have shown that reducing mutant huntingtin aggregation is associated with alleviated neuronal dysfunction and improved behavioural outcomes in HD models.^28,29^ Western blot and immunofluorescence staining analyses confirmed the increased expression of dopamine- and cAMP-regulated phosphoprotein, 32 kDa (DARPP-32) in the striatum of R6/2 mice treated with berbamine hydrochloride (Fig. 3G and Supplementary Fig. 5A and B), indicating a protective effect on neuronal signalling pathways. DARPP-32 is a key regulator of dopaminergic signalling, and its depletion is linked to motor deficits in HD.^30^ Furthermore, immunostaining revealed a significant downregulation of Gal3 and a microglial activation marker (Cd68) in the striatum of R6/2 mice treated with berbamine hydrochloride (Fig. 3H). This finding aligns with our previous study, which demonstrated that the knockdown of Gal3 in the striatum of R6/2 mice reduced the protein level of Cd68.^9^

Previous studies have demonstrated that berbamine hydrochloride can directly modulate protein conformational states and inhibit amyloid-β aggregation.^31^ We found that it did not directly alter mutant huntingtin aggregation in Neuro-2a cells expressing 109Q-HTT_Ex1_-GFP (Supplementary Fig. 5C–E). This suggests that the reduction in EM48-positive aggregation observed in R6/2 mice is more likely to be mediated through indirect mechanisms, particularly via suppression of Gal3-associated microglial activation.

### Transcriptomic profiling reveals molecular targets of berbamine hydrochloride

To investigate the effects of berbamine hydrochloride at the late stage of HD, RNA sequencing was conducted on striatal tissue collected at 13.5 weeks of age, representing the end-stage of disease progression. Principal component analysis revealed clear separation between treatment groups, demonstrating a significant transcriptional response to berbamine hydrochloride (Fig. 4A). To obtain a network-level view of transcriptional changes induced by berbamine hydrochloride, we performed weighted gene co-expression network analysis, which defined 14 gene co-expression modules that were correlated with experimental conditions to determine their biological relevance (Fig. 4B and Supplementary Table 1). This analysis groups genes into co-expression modules and assesses their correlation with each experimental condition. Among these, the blue, brown, pink and yellow modules showed strong positive correlation with the R6/2 + vehicle group (R6/2 + vec; Fig. 4B), suggesting that these modules are associated with the pathological state of HD. Intriguingly, in R6/2 mice treated with berbamine hydrochloride (R6/2 + B), the blue and brown modules lost their significant positive correlation, while the pink and yellow modules exhibited a shift to negative correlations (Fig. 4B). This suggests that berbamine hydrochloride might exert therapeutic effects by modulating the activity of these gene networks. The blue module is the largest module, containing 6167 genes, showing a high correlation of 0.79 with the R6/2 + vec group (Fig. 4B). This correlation underscores its central role in the transcriptional landscape of HD pathology. Heat maps of the blue module and its hub genes further revealed distinct expression patterns between treatment groups (Fig. 4C and D). Hub genes are highly connected genes that represent key regulatory nodes within the network. A total of 2918 hub genes were identified in the blue module (Fig. 4D). Importantly, Gal3 (encoded by *Lgals3*) was identified as a hub gene with high connectivity and significance in the blue module. A total of 2295 genes (37.2%) in the blue module showed expression patterns that were correlated with *Lgals3* across experimental conditions, indicating their inclusion in a Gal3-associated co-expression network. The co-expression network showed the top 50 Gal3-coexpressed genes (Fig. 4E and Supplementary Table 2). Intriguingly, in the berbamine hydrochloride-treated R6/2 mice (R6/2 + B; Fig. 4B), the blue module was no longer significantly correlated, consistent with modulation of Gal3-associated transcriptional networks by berbamine hydrochloride.

**Figure 4:**
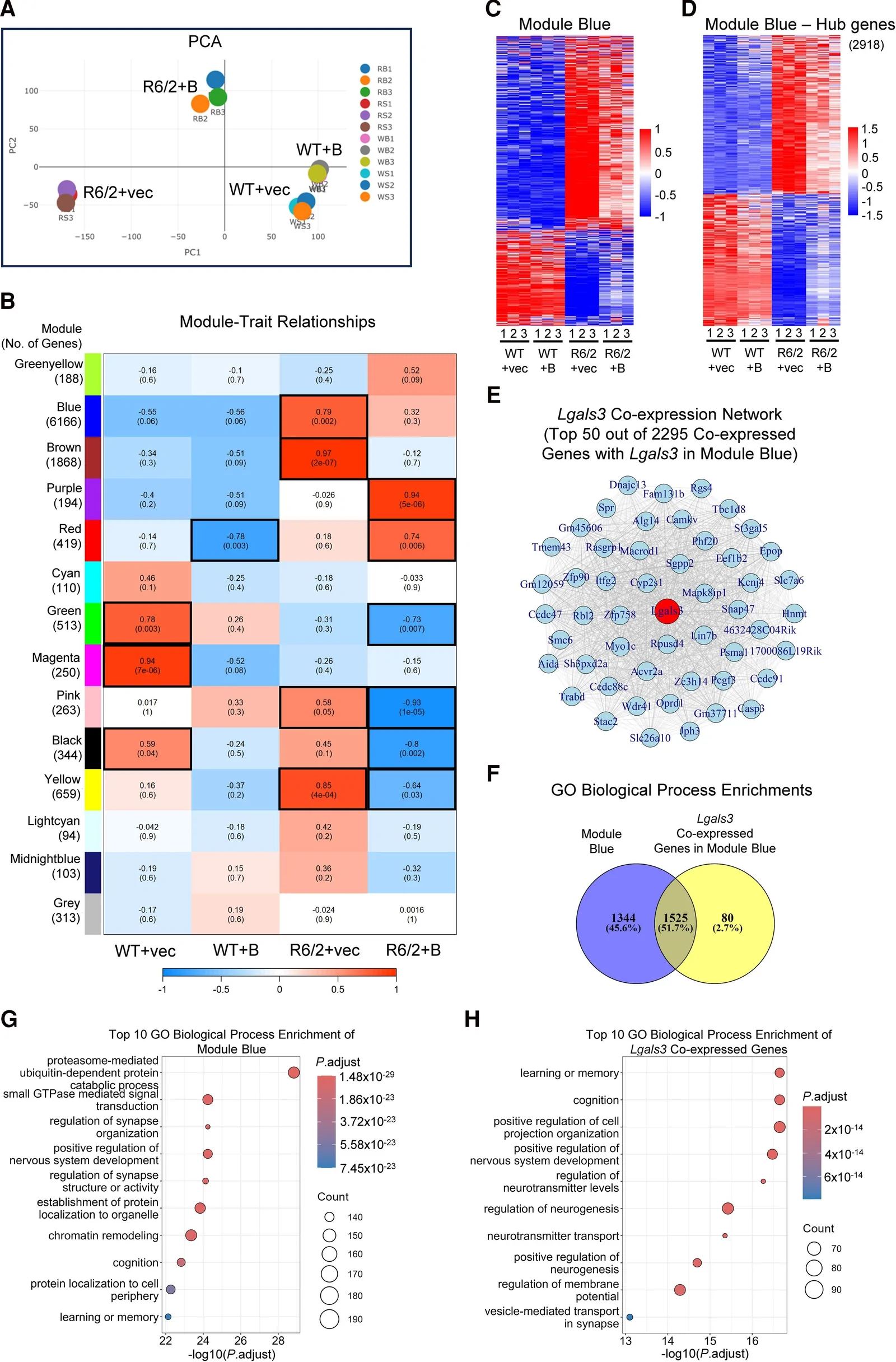
**Transcriptomic analysis of striatal brain tissue collected from R6/2 mice and WT mice treated with berbamine hydrochloride and control.** (**A**) Principal component analysis (PCA) plot showing separation between different treatment groups, WT treated with vehicle (WT + vec), WT treated with berbamine hydrochloride (WT + B), R6/2 treated with vehicle (R6/2 + vec) and R6/2 treated with berbamine hydrochloride (R6/2 + B). Each point represents one sample (*n* = 3 per group). (**B**) Module–trait relationship heat map from weighted gene co-expression network analysis (WGCNA). Each row represents 1 of the 14 gene co-expression modules (groups of genes with similar expression patterns), and each column represents an experimental condition. Each box includes the correlation coefficient and *P*-value (in parentheses). Modules with significant correlations (*P* < 0.05) are outlined in black. The module size (number of genes) is displayed on the *left*. Positive and negative correlations are shown in red and blue, respectively. (**C**) Heat map of gene expression in the blue module across different treatment groups. (**D**) Heat map of hub genes in the blue module, showing expression differences between treatment groups. (**E**) Network representation of the top 50 co-expressed genes with Gal3 (*Lgals3*) in the blue module, highlighting Gal3 as a central hub. (**F**) Venn diagram showing the overlap between Gene Ontology (GO) biological processes enriched in the blue module and Gal3 co-expressed genes within the blue module. (**G**) Top 10 GO biological process enrichments of genes in the blue module. (**H**) Top 10 GO biological process enrichments of Gal3 co-expressed genes in the blue module. B = berbamine hydrochloride; Gal3 = galectin-3; WT = wild-type.

Further functional enrichment analysis revealed that the blue module harbours 2869 enriched biological processes, including those crucial for neuronal functions, such as proteasome-mediated ubiquitin-dependent protein catabolism, synapse-related pathways, nervous system development, cognition, learning and memory (Fig. 4F and G). Among these pathways, 52% (i.e. 1525 pathways) were also enriched in the Gal3 co-expressed genes (Fig. 4F), reinforcing the functional significance of Gal3 in regulating HD-related molecular processes. Among the top 10 GO biological process enrichments, the common pathways include learning or memory, cognition and positive regulation of nervous system development (Fig. 4H). Beyond the blue module, we observed significant enrichment of biological processes in the brown and yellow modules (Fig. 4 and Supplementary Fig. 6). The brown module, containing 1868 genes, showed enrichment for pathways related to nucleotide biosynthesis and metabolism, RNA processing and cilium assembly, processes that are crucial for maintaining cellular homeostasis and regulation of gene expression (Supplementary Fig. 6A and B). The yellow module, comprising 659 genes, was enriched for pathways involved in ATP biosynthesis, oxidative phosphorylation and cellular respiration, processes crucial for energy production and mitochondrial function (Supplementary Fig. 6C and D). These findings suggest that berbamine hydrochloride not only modulates neuronal and synaptic pathways but also impacts broader metabolic and transcriptional processes crucial to HD pathology. The pink module (263 genes) did not exhibit significant enrichment for GO biological processes, suggesting a less pronounced functional role or lack of sufficient annotation.

In addition, we performed K-means clustering of the 3000 most variable genes, defined as those with the highest standard deviation in transformed expression values across all samples, found in the striatum of wild-type (WT) and R6/2 mice treated with or without berbamine hydrochloride, and defined four clusters as follows: Cluster A contains 1334 genes; Cluster B contains 329 genes; Cluster C contains 694 genes; and Cluster D contains 643 genes (Supplementary Fig. 7A and Supplementary Table 3). A heat map revealed that the genes in Cluster A and B are highly expressed, whereas genes in Cluster C are little expressed in the R6/2 + vec group. Intriguingly, treatment with berbamine hydrochloride ameliorated the gene expression in Clusters A, B and C (Supplementary Fig. 7A). No specific pattern was observed in Cluster D. Subsequent GO analysis further identified distinct biological processes associated with these clusters. Cluster A showed enrichment in processes related to microtubule cytoskeleton organization, cilium organization and cellular component assembly. Cluster B was enriched mainly in response to different stimuli processes, including interferon-beta, interferon alpha and virus. Cluster C demonstrated enrichment in processes related to neuronal function, including cell communication, synaptic signalling and behaviour (Supplementary Fig. 7B). These results are consistent with and complement the prior analyses (Fig. 4), demonstrating that Gal3 resides within transcriptional networks linked to multiple biological processes altered in HD, and that berbamine hydrochloride modulates these disease-associated gene programmes. Although Gal3 inhibition is likely to be an important contributor, these data do not exclude additional Gal3-independent mechanisms underlying the broader effects of berbamine hydrochloride.

### Berbamine hydrochloride reversed the expression of many Gal3-associated genes in HD mice

We next performed a differential gene expression analysis to assess further the roles of berbamine hydrochloride at the end stage of HD progression. Volcano plots of the differential expression analysis demonstrated significant changes in gene expression in the R6/2 + vec versus WT + vec and R6/2 + B versus R6/2 + vec comparisons (Fig. 5A and B and Supplementary Table 4). A total of 5778 differentially expressed genes (DEGs), with 4168 showing upregulation and 1610 showing downregulation, were identified in the R6/2 + vec versus WT + vec comparison group (Fig. 5A). In the R6/2 + B versus R6/2 + vec comparison, a total of 2542 DEGs were identified, with 706 showing upregulation and 1836 showing downregulation.

**Figure 5:**
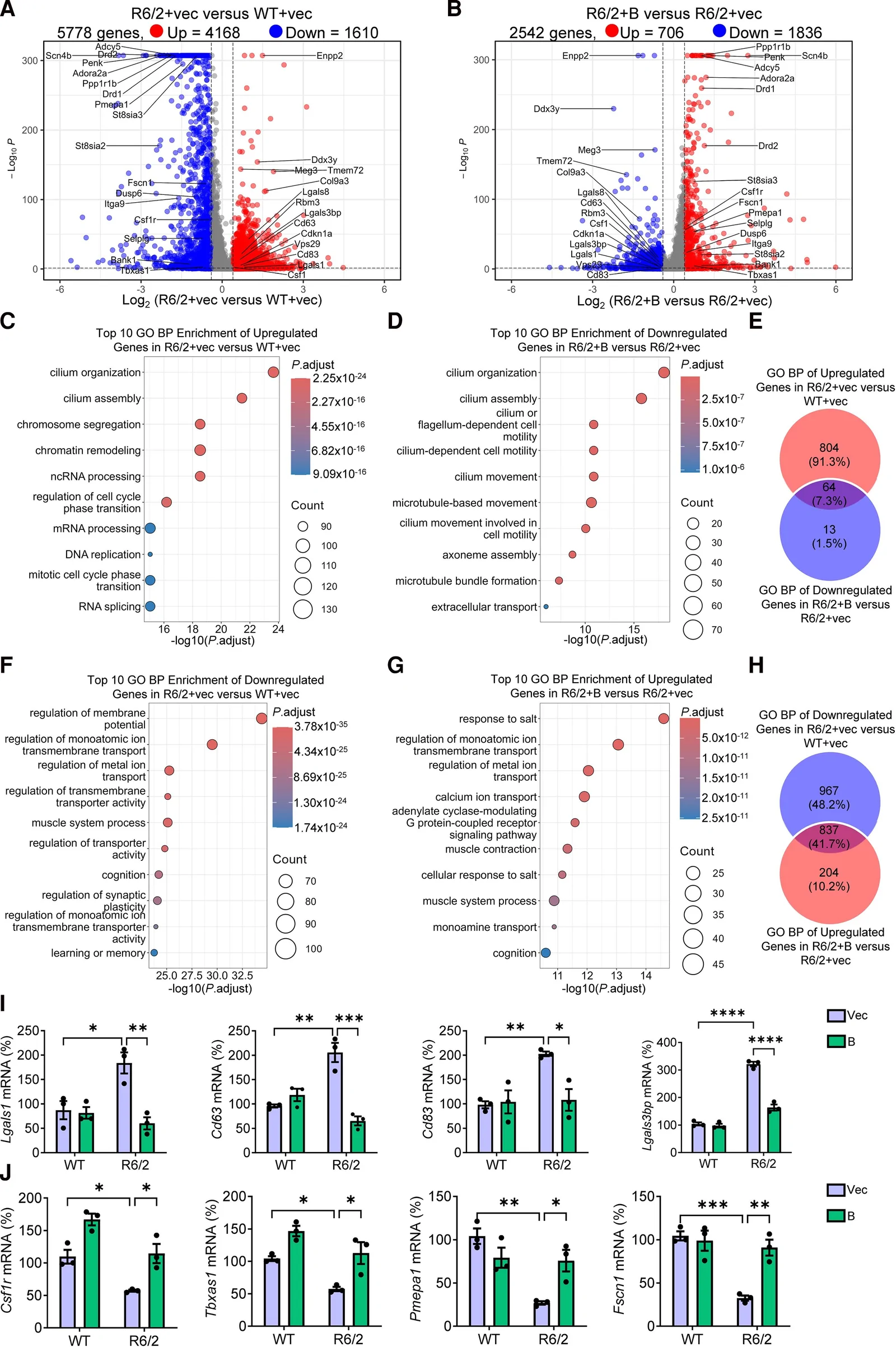
**Differential gene expression analysis and validation of selected genes in striatal brain tissue of R6/2 and WT mice treated with berbamine hydrochloride.** (**A**) Volcano plot showing DEGs in R6/2 + vec versus WT + vec. A total of 5778 genes were significantly differentially expressed, with 4168 upregulated (red) and 1610 downregulated (blue). (**B**) Volcano plot showing DEGs in R6/2 + B versus R6/2 + vec, with 2542 genes significantly differentially expressed, including 706 upregulated (red) and 1836 downregulated (blue). (**C** and **D**) Top 10 Gene Ontology (GO) biological process enrichments of upregulated genes in R6/2 + vec versus WT + vec (**C**) and downregulated genes in R6/2 + B versus R6/2 + vec (**D**). (**E**) Venn diagrams showing the overlap of GO biological processes for upregulated and downregulated genes in R6/2 + vec versus WT + vec and R6/2 + B versus R6/2 + vec, indicating how many DEGs were rescued by berbamine hydrochloride treatment. (**F** and **G**) Top 10 GO biological process enrichments of downregulated (**F**) genes in R6/2 + vec versus WT + vec and upregulated (**G**) genes in R6/2 + B versus R6/2 + vec. (**H**) Venn diagrams showing the overlap of GO biological processes for downregulated and upregulated genes, highlighting how many dysregulated biological processes were rescued by berbamine hydrochloride treatment. (**I**) Validation of selected genes that were upregulated in R6/2 + vec versus WT + vec and downregulated in R6/2 + B versus R6/2 + vec by qPCR, including *Lgals1*, *Cd63*, *Cd83* and *Lgals3bp*, *n* = 3. (**J**) Validation of selected genes that were downregulated in R6/2 + vec versus WT + vec and upregulated in R6/2 + B versus R6/2 + vec by qPCR, including *Csf1r*, *Tbxas1*, *Pmepa1* and *Fscn1* in striatal tissue from WT and R6/2 mice, *n* = 3. Data represent the mean ± standard error of the mean, analysed by two-way ANOVA with Tukey’s test; *P < 0.05, **P < 0.01, ***P < 0.001 and ****P < 0.0001. B = berbamine hydrochloride; DEGs = differentially expressed genes; Gal3 = galectin-3; WT = wild-type.

Further GO enrichment analysis showed that the upregulated genes in the R6/2 + vec versus WT + vec group include biological processes related to cilium organization, chromatin remodelling and RNA splicing (Fig. 5C). Interestingly, the downregulated genes in the R6/2 + B versus R6/2 + vec group were enriched for multiple processes related to cilium organization, microtubule-based movement and extracellular transport (Fig. 5D). Berbamine hydrochloride treatment ameliorated 7% of the biological processes enriched by the upregulated DEGs in the R6/2 + vec versus WT + vec group (Fig. 5E). For the downregulated DEGs in the R6/2 + vec versus WT + vec comparison group, the biological processes were related primarily to neuronal function, such as the regulation of membrane potential and metal ion transport, cognition, synapse, learning or memory (Fig. 5F), and berbamine hydrochloride treatment ameliorated multiple processes (42%), including the regulation of metal ion transport, monoatomic ion transmembrane transport and cognition (Fig. 5G and H). The findings from the DEG analyses are consistent with and strengthen the results of the co-expression and K-means clustering analyses (Fig. 4 and Supplementary Fig. 7).

Extensive transcriptomic studies have previously established that R6/2 mice exhibit widespread molecular dysregulation at late disease stages, particularly affecting synaptic signalling, neuronal identity genes, neurotransmitter pathways and metabolic processes, with marked downregulation of striatal medium spiny neuron identity genes, such as *Drd1*, *Tac1* and *Ppp1r1b*.^32-35^ Building on this existing knowledge, the observed changes in gene expression provide crucial insights into late-stage molecular dysfunction and the potential rescue effect by berbamine hydrochloride. Importantly, Venn diagrams indicated that berbamine hydrochloride rescued the biological processes dysregulated in R6/2 mice (Fig. 5E and H), suggesting a potential reversal of late-stage pathological changes. As a control, berbamine hydrochloride treatment in WT mice resulted in a total of 402 DEGs, with 107 showing upregulation and 295 showing downregulation (Supplementary Fig. 8A). These DEGs did not show significant enrichment in any GO biological processes, similar with a previous study showing that treatment with TD139 (a Gal3 inhibitor) did not yield significant enrichment of any biological processes in control microglia.^36^

Cell type deconvolution using Bisque revealed that DEGs affected by berbamine hydrochloride were linked to multiple cell types, including neurons, microglia, oligodendrocytes, oligodendrocyte progenitor cells, astrocytes, endothelial cells and pericytes (Supplementary Fig. 8B and C). This suggests that berbamine hydrochloride modulates transcriptional programmes across diverse brain cell populations. Similar to previous studies implicating Gal3 in neuroinflammatory processes that impact multiple cell types, Gal3 inhibition might contribute to these widespread effects, although additional Gal3-independent pathways are also likely to be involved.^36,37^

We have previously identified Gal3-associated microglial genes in a mouse model of tauopathy by single-cell RNA sequencing.^36^ Although bulk RNA sequencing reflects transcriptomic profiles from all cell types, we focused on a subset of genes previously identified as Gal3-associated microglial genes. To assess the effect of berbamine hydrochloride on Gal3 inhibition, we selected seven upregulated DEGs in the R6/2 versus WT comparison, which were downregulated in the R6/2 + B versus R6/2 + vec comparison, and also identified as Gal3-associated microglial genes in prior study, for quantitative real-time PCR (RT-qPCR) validation. These genes included *Lgals1*, *Cd63*, *Cd83*, *Rbm3*, *Vps29*, *Cdkn1a* and *Csf1*, with *Lgals3bp* also included as our gene of interest. Except for *Csf1*, the seven selected DEGs were successfully validated in the qPCR analysis (Fig. 5I and Supplementary Fig. 9A). Likewise, we selected eight downregulated DEGs in the R6/2 versus WT comparison, upregulated in the R6/2 + B versus R6/2 + vec comparison, and also previously identified as Gal3-associated microglial genes for qPCR validation. These genes included *Csf1r*, *Tbxas1*, *Pmepa1*, *Fscn1*, *Dusp6*, *Bank1*, *ITga9* and *Selpl9*. Six of the genes were successfully validated. The expression patterns of *Bank1*, *Itga9*, *Selplg* and *Csf1* aligned with the RNA sequencing analysis, although statistical significance was not achieved (Fig. 5J and Supplementary Fig. 9B), possibly owing to the limited sample size. The results strengthen the role of berbamine hydrochloride in the inhibition of Gal3.

### Therapeutic effects of berbamine hydrochloride on microglia and beyond in HD mice

From the DEG analysis, we found that *Lgals1*, the gene coding for galectin-1 (Gal1), another member of the galectin family, which is also a Gal3-associated microglial gene^36^ and a marker for microglial activation in the ageing brain,^38^ was upregulated in R6/2 mice. Treatment with berbamine hydrochloride normalized this abnormal upregulation of Gal1. Immunofluorescence (IF) staining revealed that these changes occurred in the corpus callosum region, which is located between the striatum and cortex (Fig. 6A and B). Importantly, most Gal1-positive cells were also positive for Iba1 (a microglia marker) staining, indicating that the upregulation of Gal1 occurs in microglia. Berbamine hydrochloride treatment significantly reduced Gal1 levels in R6/2 mice (Fig. 6A and B).

**Figure 6:**
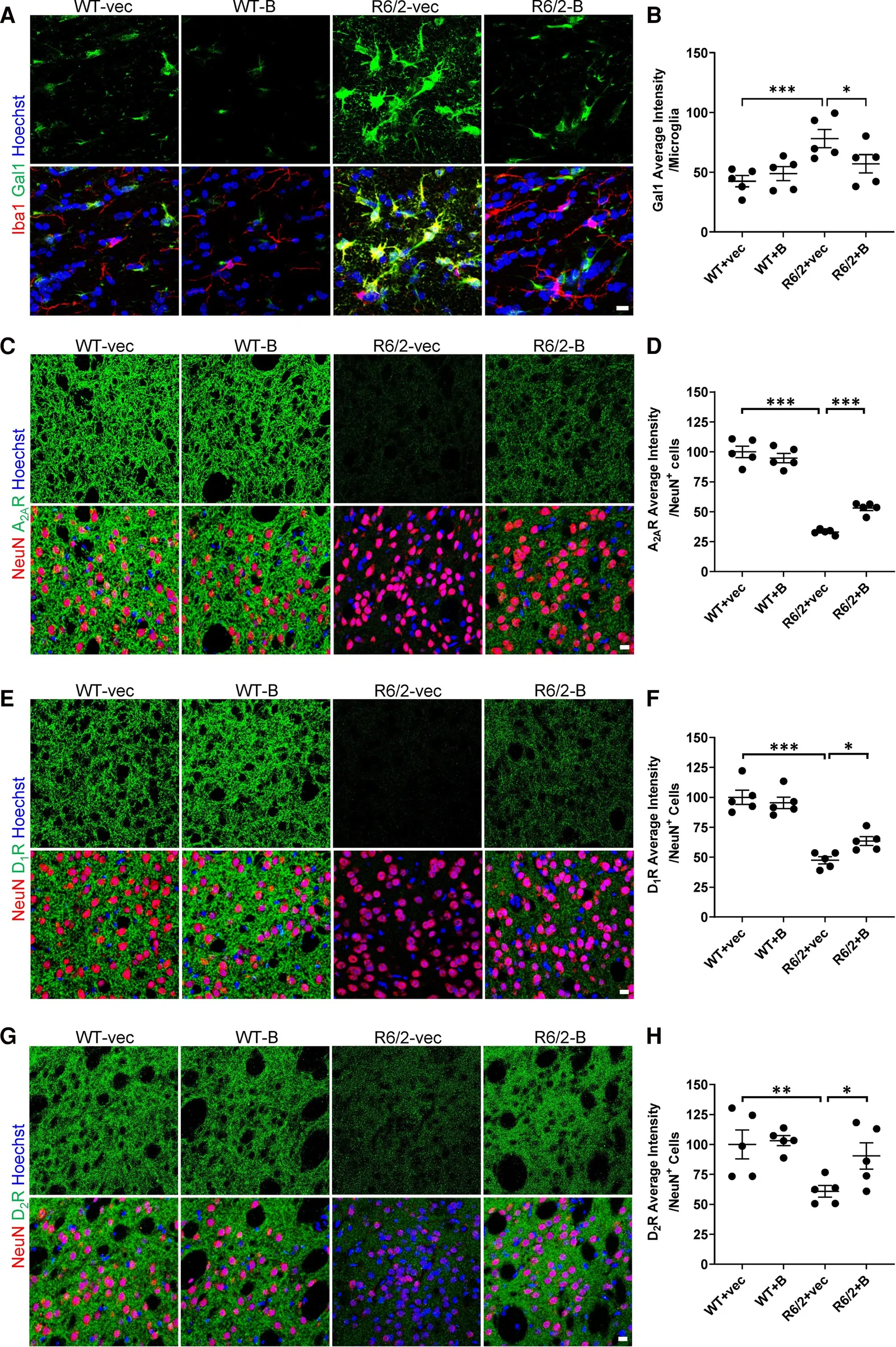
**Effects of berbamine hydrochloride treatment on Gal1, adenosine A_2A_ receptor and dopaminergic receptors in the brain of R6/2 mice.** (**A**) Immunofluorescence staining showing Gal1 (green) and Iba1 (red) in the corpus callosum. Hoechst staining (blue) was used to label nuclei. (**B**) Quantification of Gal1 expression levels in microglia across treatment groups (*n* = 5). (**C**) Immunofluorescence staining showing A_2A_ adenosine receptor (A_2A_R, green) and NeuN (red) in the striatum. Hoechst staining (blue) was used to label nuclei. (**D**) Quantification of A_2A_R expression levels in NeuN-positive cells across treatment groups, *n* = 5. (**E**) Immunofluorescence staining showing dopamine receptor D_1_R (green) and NeuN (red) in the striatum. Hoechst staining (blue) was used to label nuclei. (**F**) Quantification of D_1_R expression levels in NeuN-positive cells across treatment groups, *n* = 5. (**G**) Immunofluorescence staining showing the dopamine receptor D_2_R (green) and NeuN (red) in the striatum. Hoechst staining (blue) was used to label nuclei. (**H**) Quantification of D_2_R expression levels in NeuN-positive cells across treatment groups, *n* = 5. Data are presented as the mean ± standard error of the mean and analysed by two-way ANOVA with Tukey’s test; *P < 0.05, **P < 0.01 and ***P < 0.001. Scale bars = 10 µm. B = berbamine hydrochloride; vec = vehicle; WT = wild-type.

GO enrichment analysis highlighted processes, such as the regulation of metal ion transport, calcium ion transport and adenylate cyclase-modulating G-protein-coupled receptor signalling, that were rescued by treatment with berbamine hydrochloride (Fig. 5G). More than 40 genes are implicated in these GO terms, including *Adora2a*, *Drd1* and *Drd2*, which are known to be downregulated in HD. In particular, A_2A_R (*Adora2a*) is involved in modulating neurotransmitter release and has been linked to neuroprotective effects, while both D_1_R (*Drd1*) and D_2_R (*Drd2*) play critical roles in the direct and indirect pathways of dopaminergic signalling, which are disrupted in HD.^39^ In addition, A_2A_R/D_2_R and D_1_R are enriched in medium spiny neurons in the striatum and serve as markers for the indirect and direct pathways.^40^ To assess whether treatment with berbamine hydrochloride also impacts the degeneration of the striatal pathways, IF assays were conducted to validate the rescue effect of berbamine hydrochloride. Results of IF staining showed significant downregulation of A_2A_R in HD mice compared with the WT mice, and treatment with berbamine hydrocholoride significantly reversed the loss of A_2A_R in HD mice (Fig. 6C and D). Similar patterns were observed for the expression of D_1_R (Fig. 6E and F) and D_2_R (Fig. 6G and H), confirming the results from transcriptomic analyses.

Dysregulation of ciliogenesis has been implicated in HD,^41^ although its relationship to Gal3 remains underexplored. Interestingly, berbamine hydrochloride treatment reduced the enrichment of biological processes related to cilium organization in the late stage of R6/2 mice (Fig. 5D). To validate the effects of berbamine hydrochloride on ciliogenesis, we performed immunofluorescence staining, which revealed an increase in the average length of cilia, as indicated by the levels of the cilia marker Arl13b in astrocytes of R6/2 mice. This increase was attenuated by berbamine hydrochloride treatment (Fig. 7A and B), suggesting that the therapeutic effects of berbamine hydrochloride might extend beyond Gal3 inhibition in microglia to influencing astrocytic ciliogenesis.

**Figure 7:**
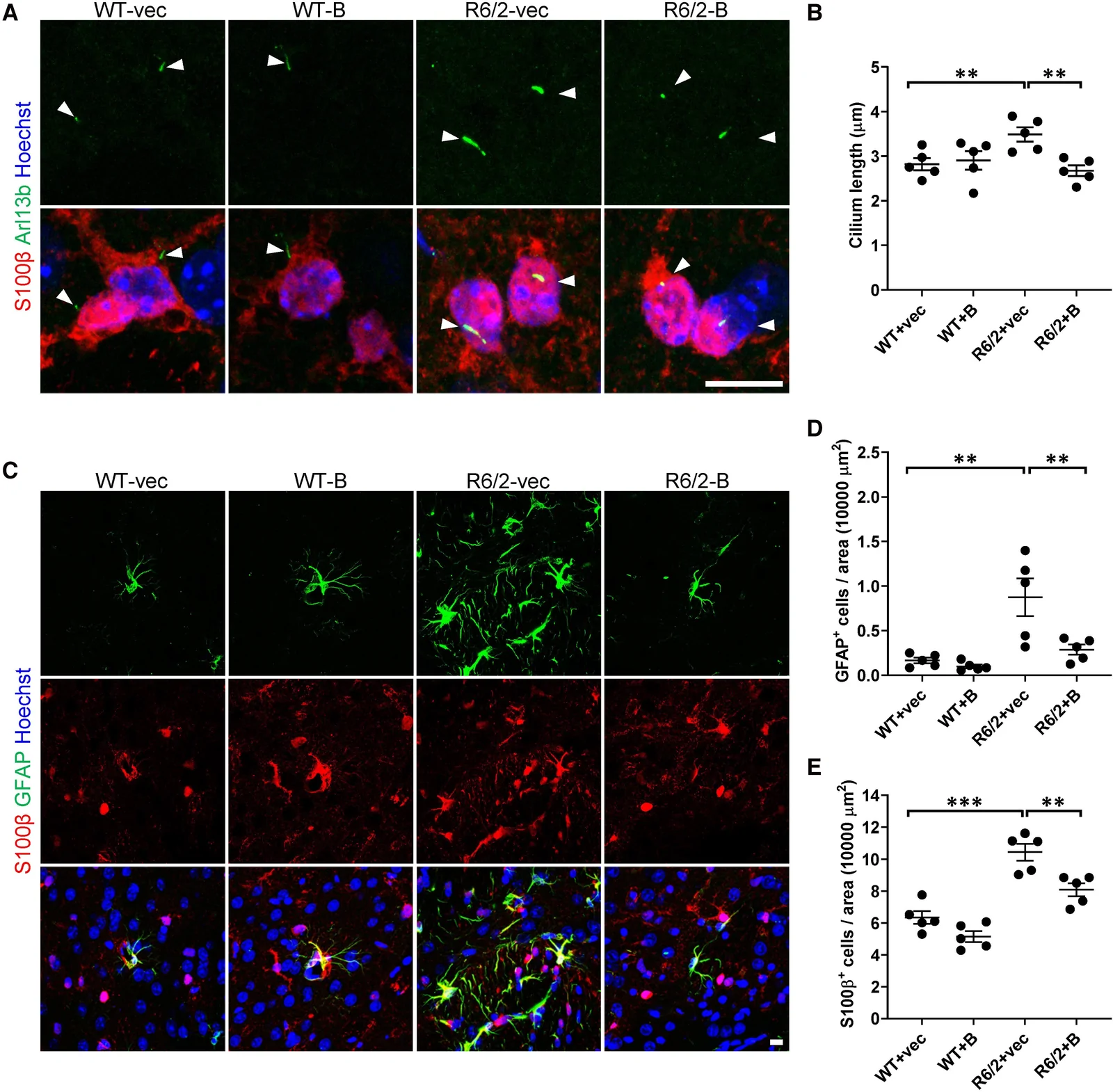
**Effects of berbamine hydrochloride treatment on astrocytes in R6/2 mice.** (**A**) Immunofluorescence staining showing cilia (Arl13b, green) and astrocytes (S100β, red) in the striatum. Arrows indicate the presence of cilia on astrocytes. (**B**) Quantification of cilia in astrocytes across treatment groups, *n* = 5. (**C**) Immunofluorescence staining of GFAP (green) and astrocytes (S100β, red) in the striatum. (**D** and **E**) Quantification of GFAP-positive astrocytes and total astrocytes across treatment groups, *n* = 5. Data are presented as the mean ± standard error of the mean and analysed by two-way ANOVA with Tukey’s test; **P < 0.01 and ***P < 0.001. Scale bars = 10 µm. B = berbamine hydrochloride; GFAP = glial fibrillary acidic protein; vec = vehicle; WT = wild-type.

To assess whether berbamine hydrochloride also modulate astrocytic features, we next examined the expression levels of S100β, a pan-astrocytic marker, and glial fibrillary acidic protein (GFAP), a canonical marker of reactive astrocytes.^42^ Immunofluorescence staining revealed a pronounced elevation of GFAP-positive astrocytes in the striatum of R6/2 mice compared with WT controls (Fig. 7C). Notably, berbamine hydrochloride treatment significantly reduced the density of both GFAP-positive and S100β-positive astrocytes in R6/2 mice (Fig. 7D and E). Together, these results demonstrate that long-term berbamine hydrochloride treatment attenuates aberrant astrocytic reactivity in the HD brain.

## Discussion

Recent advances in HD therapeutics underscore the clinical urgency and translational relevance of identifying complementary disease-modifying strategies. Notably, uniQure recently reported that its gene therapy, AMT-130, achieved ∼75% slowing of disease progression over 3 years in a phase 1/2 clinical trial of early-stage HD patients.^43^ This approach suppresses huntingtin expression via microRNA-mediated RNA interference, directly targeting the genetic cause of HD. However, such gene-silencing strategies require invasive intracerebral delivery and reduce both mutant and wild-type huntingtin. These considerations highlight the need to identify additional therapeutic targets for the development of new treatments for HD.

The identification of Gal3 as a therapeutic target has garnered considerable attention owing to its established role in neurodegenerative diseases.^3^ In HD, Gal3 is a crucial mediator of microglial activation, in which the accumulation of Gal3 in the damaged lysosomes inhibits their effective clearance, ultimately driving the pro-inflammatory responses and neuronal damage.^9^ Our previous work has demonstrated that genetic suppression of Gal3 in the R6/2 HD mouse model significantly improves motor deficits, directly establishing Gal3 as a disease-modifying factor in HD.^9^ Additionally, Gal3 has been shown to play a role in AD.^36,44,45^ Gal3 was upregulated in microglia in human and mouse models of tauopathy, such as AD, where it promoted the accumulation of pathogenic tau and enhanced tau fibrillation.^36^ Genetic removal of Gal3 in a tauopathy mouse model suppressed microglial activation, reduced pTau levels and rescued memory impairment.^36^ Moreover, Gal3 is upregulated in microglia upon exposure to different forms of α-synuclein, whereas inhibition of Gal3 has led to reduced inflammatory cytokine production and phagocytic activity in models of PD.^46^ These findings underscore the potential of targeting Gal3 in multiple neurodegenerative disorders.

In this study, we have successfully identified and validated berbamine hydrochloride as a potent inhibitor of Gal3 through a high-content screening approach using a stable microglial cell line expressing Gal3-GFP. The accumulation of Gal3 into puncta, induced by lysosomal damage via LLOMe, served as a reliable indicator of intracellular glycan-dependent Gal3 activity.^47^ To place the diversity of inhibitory profiles observed in our screen in context, it is worth noting that initial hits, including Z-FA-FMK (a cysteine protease inhibitor widely used to block cathepsin activity), multiple calpain inhibitors, ryuvidine (a cyclin-dependent kinase inhibitor), TX-1123 (an antitumour tyrosine kinase inhibitor) and valtrate (a plant-derived bioactive compound with antiviral and antitumour properties), also reduced Gal3 puncta formation.^48-51^ Although these compounds were not pursued further, their known biochemical activities suggest that multiple upstream pathways might modulate Gal3 recruitment to damaged lysosomes. To enhance the reliability of our screening results and minimize the risk of false positives, we incorporated a direct binding inhibition assay alongside the cell line-based screening. By using an HTRF assay, we assessed the direct interaction between Gal3 and its glyco-ligands, such as TREM2 and between Gal3 itself.^4,20^ This was crucial to ensure that the observed reduction in Gal3-GFP puncta was indeed attributable to inhibition of Gal3 activity, rather than off-target effects or alterations in the cellular environment that could influence the readout. For instance, compounds that affect the availability of β-galactoside residues, which are recognized by Gal3, could lead to misleading interpretations of their apparent inhibitory effects.^52^ By validating our findings through direct binding inhibition assays, we could confidently attribute the observed reductions in Gal3 puncta formation to the specific inhibition of Gal3.

When designing our screening models, we applied full-length Gal3 in both the cell model and the HTRF assay. Full-length Gal3 is essential for the oligomerization and functional properties of the protein, because it retains both the CRD and the NTD, which are crucial for its biological activities. Previous study indicates that the NTD plays a vital role in ligand-dependent oligomerization, which is not achievable with the CRD alone.^53^ This oligomerization enhances the avidity of Gal3 interactions with multivalent glyco-ligands, compared with the isolated CRD.^54^ Furthermore, studies have shown that the full-length protein is necessary for high-affinity interactions with microbial and mammalian glycans, highlighting its importance in immune responses and pathogen interactions.^54,55^ In contrast, truncated forms, such as Gal3C, which lack the NTD, are unable to form higher-order oligomers and exhibit diminished functional capabilities, such as impaired angiogenic responses.^56^ The oligomeric state of full-length Gal3 is crucial for its role in cellular signalling and receptor clustering, which are pivotal for its participation in various physiological processes, including inflammation and immune modulation.^57,58^ Earlier study has reported that full-length Gal3 promotes the aggregation of amyloid-β.^44^ Intriguingly, a recent study demonstrates that the full-length Gal3 facilitates the fibrillation of misfolded tau, whereas the CRD or the NTD alone has no such effect.^36^ Therefore, the use of full-length Gal3 in the screening is pivotal to identify a potent inhibitor. The identification of berbamine hydrochloride, which shows inhibitory effects on the full-length Gal3, highlights its potential therapeutic role in modulating Gal3-related pathways. Berbamine hydrochloride is likely to inhibit Gal3 puncta formation by interfering with its oligomerization and conformational state. Rather than directly competing at the CRD, the compound might act through allosteric modulation, binding to a site distal to the CRD, yet inducing structural changes that impair the glycan-binding activity of Gal3. This mechanism is reminiscent of PTX-008, an angiostatic agent shown by nuclear magnetic resonance studies to bind the non-CRD region of Gal3, altering its conformation and ultimately attenuating CRD function.^25^ Such allosteric inhibition could underlie the efficacy of berbamine hydrochloride in reducing Gal3 puncta and modulating its downstream effects, representing an avenue warranting further structural investigations beyond the scope of the present study.

Berbamine hydrochloride, a compound derived from the traditional Chinese medicinal herb *Berberis amurensis*,^59^ has been reported previously to possess various pharmacological effects, including anti-inflammatory, anticancer, calcium channel antagonist, calpain regulatory and antiviral properties, such as inhibition of SARS-CoV-2 and Ebola virus infection.^59-62^ Zhang *et al*.^60^ reported that berbamine hydrochloride potently inhibits SARS-CoV-2 infection by blocking S protein-mediated membrane fusion. Likewise, Yi *et al*.^59^ demonstrated its efficacy against the Ebola virus by targeting viral glycoproteins, further supporting its potential as a broad-spectrum antiviral agent. Recent studies have highlighted berbamine hydrochloride for reducing amyloid-β deposition and improving learning and memory in mouse models of AD via oral administration.^62^ Notably, even at a high dose of 280 mg/kg/day administered by gavage for 40 days, berbamine hydrochloride showed no adverse effects, supporting its safety profile. Additionally, in a study by Dong *et al*.,^31^ berbamine hydrochloride was shown to inhibit amyloid-β aggregation and cytotoxicity effectively in neuronal cells. Interestingly, Gal3 has been implicated in these conditions, and the therapeutic effects of berbamine hydrochloride observed in these previous studies might be attributed, in part, to its inhibition of Gal3. However, given its diverse pharmacological activities, berbamine hydrochloride is likely to exert its effects through multiple mechanisms. Although Gal3 inhibition is a significant factor, it is also possible that berbamine hydrochloride targets additional pathways, contributing to its overall therapeutic efficacy.

In the context of HD, these pleiotropic actions are particularly relevant for neuronal circuits governing motor control, which are highly sensitive to neuroinflammatory modulation. The activation of Drd1 in the direct pathway facilitates movement and action initiation, whereas Drd2 in the indirect pathway inhibits competing motor programmes, highlighting their opposing functions in motor control.^63,64^ Dysregulation of these receptors in HD leads to impaired motor coordination and other movement-related symptoms owing to the imbalance between these pathways. The therapeutic inhibition of Gal3 by berbamine hydrochloride is likely to reverse the downregulation of these receptors indirectly, through microglia–neuron interactions (Fig. 6E–H), thereby improving neuronal function and mitigating the progression of HD by restoring normal signalling pathways.

Transcriptomic analysis further demonstrated the efficacy of berbamine hydrochloride at the end stage of HD progression. The identification of 14 gene co-expression modules using WGCNA revealed that berbamine hydrochloride treatment led to significant changes in gene expression in the R6/2 mouse model. To benchmark our findings against existing preclinical HD datasets, we compared the DEGs regulated by berbamine hydrochloride in R6/2 mice with the curated gene expression datasets from the CHDI HDinHD Preclinical Informatics Resource (https://chdifoundation.org/preclinical-informatics-hdinhd/). Berbamine hydrochloride reversed a portion of disease-associated changes identified across independent R6/2 striatal datasets, supporting the translational relevance of berbamine hydrochloride (Fig. 5C–H).

The role of Gal1 in HD pathology is also of significant interest. Besides being another member of the galectin family, Gal1 was previously identified as a Gal3-associated microglial gene in a mouse model of tauopathy, namely THY-Tau22.^36,65^ Here, we found that Gal1 is upregulated at both the transcript and protein levels in R6/2 mice, whereas it is downregulated upon berbamine hydrochloride treatment. Our findings, in concordance with existing literature, suggest that Gal1-expressing microglia might play a significant role in regulating neuroinflammation associated with ageing. In particular Gal1 expression selectively marks microglial populations associated with ageing in the brain, and its expression is linked to the activated phenotype of microglial cells.^38^ This is interesting because HD has been reported to exhibit ageing acceleration.^66^ It is worth noting that we observed the presence of Gal1-expressing microglia only at the corpus callosum region. The detection of Gal1 transcript (*Lgals1*) in the RNA sequencing and qPCR studies might be attributable to inclusion of the corpus callosum during brain striatal tissue collection or might simply reflect the presence of the transcript, but not protein, in the striatal regions. The role of Gal1 in HD microglia is not fully understood and warrants future investigation.

## Conclusion

Taken together, our findings establish berbamine hydrochloride as a novel Gal3 inhibitor with the ability to cross the BBB. By effectively modulating Gal3-associated gene networks, berbamine hydrochloride demonstrates a significant capacity to delay disease progression in a mouse model of HD. These results underscore its therapeutic potential for mitigating the pathological features of HD and advancing targeted strategies for neurodegenerative diseases. Given that the overall structure of Gal3 remains unresolved, the structure-based drug design for full-length Gal3 is currently unavailable. Our screening strategy, which used cell-based Gal3-GFP puncta imaging and an HTRF assay, provided an effective method for identifying novel Gal3 inhibitors. This strategy can also be adapted for use with other galectins or for identifying galectin enhancers. Our findings provide compelling evidence that berbamine hydrochloride is a potent Gal3 inhibitor with significant therapeutic potential in neurodegenerative diseases. It is essential to acknowledge that berbamine hydrochloride is a multi-target compound, and its therapeutic effects are likely to be mediated by both Gal3-dependent and Gal3-independent mechanisms. Future studies are required to explore its long-term effects in various neurodegenerative models and to investigate its broader pharmacological profile. In addition, future studies incorporating both male and female HD mice, Gal3 knockout models and cell type-resolved transcriptomic and proteomic analyses will be important to extend the current findings across sexes, and Gal3-specific therapeutic effects of berbamine hydrochloride.

## Supplementary Material

awag069_Supplementary_Data

## Data Availability

All relevant data that support this study are provided as supplementary files or available to any interested researchers upon reasonable request to the corresponding author. RNA data SRA files have been deposited in the NCBI’s Sequence Read Archive (PRJNA1328396).
