# Supplementary material for: Berbamine hydrochloride as a brain-penetrant galectin-3 inhibitor in a model of Huntington’s disease
Source: Brain. 2026 Mar 3;149(10):3414–30. doi: 10.1093/brain/awag069 (PMC13634645; doi:10.1093/brain/awag069)
Supplement: Supplementary file 1 [file awag069_supplementary_data.zip › brain-2025-01834-File009.pdf]

Berbamine hydrochloride as a brain penetrant Galectin-3 inhibitor in a model of Huntington's disease

Jian Jing Siew[1], Chia-Wei Lee[1], Hui-Mei Chen[1], Hsien-Ya Lin[2], Yi-Ling Liu[3], Yi-Hsuan Lee[4,5], Ying-Ta Wu[3], Hung-Lin Chen[6], Fu-Tong Liu[1,7], Chun-Hung Lin[2], Yijuang Chern[1]

Author affiliations:

[1]Institute of Biomedical Sciences, Academia Sinica, Taipei 115, Taiwan

[2]Institute of Biological Chemistry, Academia Sinica, Taipei 115, Taiwan

[3]Genomics Research Center, Academia Sinica, Taipei 115, Taiwan

[4]Department and Institute of Physiology, College of Medicine, National Yang Ming Chiao Tung University, Taipei 112, Taiwan

[5]Brain Research Center, National Yang Ming Chiao Tung University, Taipei 112, Taiwan

[6]Master Program in Clinical Genomics and Proteomics, Taipei Medical University, Taipei 110, Taiwan

[7]Department of Dermatology, Keck School of Medicine, University of Southern California, Los Angeles, CA

Correspondence to: Yijuang Chern

Full address: Institute of Biomedical Sciences, Academia Sinica, Nankang, Taipei 115, Taiwan

E-mail: bmychern@ibms.sinica.edu.tw

Running title: Brain penetrant Galectin-3 inhibitor

Keywords: Neurodegeneration, Huntington's disease, Microglia, Galectin, Drug Screening

Materials and methods

Establishment of stable microglial cell line expressing Gal3-GFP

A stable microglial cell line, SM826, which was derived from spontaneously immortalized primary mouse microglia,[1] expressing Gal3 fused with GFP was generated to monitor Gal3 puncta formation. Briefly, a lentiviral construct (pLV.PGK.GFP.miR9T), gifted by Johan Jakobsson's lab,[2] were subcloned to express either Gal3-GFP or GFP as a control. SM826 cells were transduced with lentivirus at an MOI of 100 to express Gal3-GFP or GFP, respectively. After two passages, the cells were then sorted using BD FACSAria™ III Cell Sorter (BD Biosciences, USA), selecting GFP-positive cells to confirm successful transduction. Stable clones were confirmed by Western blot analysis for Gal3-GFP.

Ultra-high throughput screening for Gal3 inhibitors

The high-content screening was conducted using SM826 cells expressing Gal3-GFP (SM826-Gal3-GFP), with SM826 cells expressing GFP alone (SM826-GFP) as the control. Cells were seeded at 600 cells/well in 1536-well collagen-coated plates (#6004920, Perkin Elmer). After 24 hours of incubation, the cells were treated with a library of 23,936 compounds, including FDA-approved drug, natural products, and local chemist drug collection, at a single concentration of 10 μM. TD139 was included as a positive control. After 24 hours of compound treatment, lysosomal damage was induced by treating the cells with L-leucyl-L-leucine methyl ester (Llome, 1 mM) for 2 hours, resulting in the formation of Gal3-GFP puncta. Cells were washed with PBS, fixed with 4 % paraformaldehyde, and stained with Hoechst. The fixed cells were stored in PBS in the dark at 4°C prior to analysis using the Opera Phenix high-content screening system (Perkin Elmer, USA). In the primary screening, hits were selected based on two criteria: (1) the computed percentage of puncta-positive cells was lower than that observed in the positive control and the low puncta signal condition (Llome, 0.25 mM), and (2) the cell count was not lower than the mean cell count of the Llome-treated condition on each plate, to exclude compounds that might affect cell viability. In the secondary screening, hit compounds were re-evaluated using freshly prepared compound libraries from an independently purchased lot. These compounds were tested in an eight-point, two-fold dilution series, ranging from 10 μM to 0.078 μM. A total of 24 hits with

reproducible potency and dose-dependent IC_{50} less than 5 μM were identified, and these hits were manually inspected to eliminate false positives and compounds that caused abnormal cellular morphology.

HTRF assay for Gal3 direct binding inhibition

HTRF assay was employed to evaluate the ability of identified compounds to inhibit Gal3 interactions. Recombinant Gal3 was expressed and purified as previously described,[3] while the recombinant human TREM2, produced in HEK293 cells and thus glycosylated, was purchased from Abcam (ab271773, Abcam). The Eu cryptate labeling kit, sourced from Cisbio, and the Alexa647 labeling kit (A20173, Invitrogen) were utilized for the labeling of Gal3 and Trem2. HTRF signals for Alexa647-labeled Gal3 binding to Eu-labeled TREM2 or Eu-labeled Gal3 were measured by excitation at 317 nm, with fluorescence emission at 620 nm for the donor (Eu) and 667 nm for the acceptor (Alexa647). HTRF competition binding experiments were conducted in 384-well white Proxi plates (6008280, Perkin Elmer) with a total assay volume of 25 μl. The assay buffer provided by the Eu cryptate labeling kit was optimized for this purpose. Initially, 10 μl of 50 nM Alexa647-labeled Gal3 was added to each well and incubated with the inhibitors for 10 minutes at room temperature. The inhibitors were dissolved in DMSO and diluted to a final concentration of 2.5 mM in the assay condition, except for Calpain inhibitor XI, which had a final concentration of 1.75 mM due to its solubility. Following the incubation period, 5 μl of Eu-labeled TREM2 (final concentration 2 nM) or Eu-labeled Gal3 (final concentration 20 nM) was added to the wells. The plate was then incubated for an additional hour at room temperature before measurement using a Tecan infinite M1000Pro plate reader. Data analysis was performed using the following equation: % Inhibition = [(DMSO only (667 nm / 620 nm)) – (Inhibitor (667 nm / 620 nm))] / DMSO only (667 nm / 620 nm) X 100. This approached provided quantitative insight into the inhibitory effects of the compounds on Gal3 binding.

ELISA

ELISA was used to quantify TNFα secretion in SM826 microglial cells treated with LPS and hit compounds. Briefly, SM826 cells were pretreated with 10 μM of each compound for 24 hours, followed by replacement with fresh medium containing both 10 μM of the compounds and 50 ng/ml of LPS for an additional 24 hours. TNFα in culture medium were determined using the

mouse TNF alpha Uncoated ELISA kit (88-7324, Thermo Scientific) according to the manufacturer's instructions. The optical density at 450 nm was measured using a SpectraMax 190 microplate reader (Molecular Devices, USA), with the value at 570 nm subtracted as a wavelength correction. TNFα were calculated by reference to a standard curve prepared with the appropriate TNFα standard.

Blood-brain barrier permeability test in mouse

To evaluate the ability of hit compounds to cross the BBB, mice were intraperitoneally injected with 5 mg/kg of Berbamine hydrochloride (BCN2400, BioCrick), Z-FA-FMK (A8170, APExBIO) or vehicle controls. At the indicated time points post-injection (15 min, 30 min, 1, 2, 4, 8, and 24 hours), blood samples were first collected, Mice were then immediately transcardially perfused with saline to remove residual blood from the cerebral vasculature prior to brain collection. Plasma and brain samples without compound injection were used as negative controls and for standard curve construction. Blood samples were centrifuged at 1000 g for 10 min at room temperature, supernatants were collected and stored at -80 °C. Brain samples homogenized with Tris-buffered saline and centrifuged at 16000 g for 30 min at 4°C, with the supernatants collected and stored at -80°C. Compound concentrations in the plasma and brain homogenates were analyzed using the UPLC/MS/MS system at the Proteomics Core Facility of the Institute of Biomedical Sciences, Academia Sinica.

Animals

R6/2 (B6CBA-Tg(HDexon1)62Gpb/1J) mice were initially purchased from the Jackson Laboratory (Bar Harbor, ME, USA) and subsequently maintained as colonies in the animal facility of the Institute of Biomedical Science under standard housing conditions. HD is an autosomal dominant disorder that affects both sexes. However, male and female R6/2 mice exhibit slight differences in disease progression, with female mice generally having a slightly longer median survival compared to males.[4] To minimize potential variability due to sex differences, only male mice were used in all experimental procedures.

Motor performance and body weight assessment

Mice were i.p. injected with Berbamine hydrochloride (5 mg/kg) or vehicle control daily, from Monday to Friday, starting at 6 weeks of age until the end of the study. Rotarod testing was conducted from 7 weeks of age to evaluate motor coordination, and clasping behavior tests began at 10 weeks of age as an indicator of neurological deficits. For the rotarod assessment, mice underwent trials three days per week, with three trials per day, for a maximum duration of 120 seconds at a constant speed of 12 rpm. The highest score achieved in a day was recorded as the daily score, and the weekly score was calculated as the average of the highest daily scores from the three test days of the week. For the clasping assessment, mice were suspended by the tail for 30 seconds. Limb clasping was scored as follows: 0 for no clasping, 1 for clasping with either the fore or hind limbs for 15 seconds, and 2 for clasping with both fore and hind limbs for 15 seconds.[5] Body weight was also monitored from 7 week of age as an additional indicator of disease progression. These three assays were conducted in two independent batches by two different experimenters. This first batch involved a small pilot experiment using five mice per group showed beneficial effects of Berbamine hydrochloride at this dose, providing further confidence to proceed with the full study using this concentration. The first batch concluded at 13.5 weeks of age, while the second batch concluded at 12.5 weeks of age. The final results combined data from both batches and were analyzed up to 12.5 weeks of age.

Immunofluorescence staining

Immunofluorescence staining was performed on brain sections as previously described.[6] Briefly, mice were anesthetized via i.p. injection of 50 mg/kg Zoletil 50 (#05393, Virbac), followed by transcardial perfusion with saline. Brain tissues were harvested, fixed in 4 % paraformaldehyde for 3 days at 4°C, and equilibrated in 30 % (w/v) sucrose for 3 days. The tissues were sectioned coronally at a thickness of 20 μm and stored in 0.1 M phosphate buffer (pH 7.4) with 0.1 % sodium azide. Brain sections were permeabilized by 0.2 % (v/v) Triton X-100 and blocked in 4 % (w/v) BSA for 1 hour. Primary antibodies were incubated for 2 to 3 days at 4°C in a humidified chamber, followed by incubation with the appropriate secondary antibodies for 2 hours at room temperature. Nuclei were stained with Hoechst 33258. Primary antibodies included Iba1 (1: 500, 019-19741, Wako Laboratory Chemicals), NeuN (1: 1000, ABN78 and 1:500, MAB377, Millipore), DARPP-32 (1: 50, #2302, Cell Signaling), EM48 (1: 500, MAB5374, Chemicon), DARPP-32 (1: 50, #2302, Cell Signaling), Gal1 (1: 200, AF1245, R&D Systems), $A_{2A}R$ (1: 200, MSFR100080, Frontier

Institute), D_1R (1: 200, Af1000, Frontier Institute), D_2R (1: 200, AB_2571596, Cosmo Bio), Arl13b (1: 100, ab136648, Abcam), S100β (1: 1000, Z0311, Dako) and GFAP (1: 500, G3893, Sigma). For EM48 histological analysis, five mice per group were included. Coronal brain sections at matched anatomical levels were stained with EM48 and NeuN. Quantification was performed across the entire striatum and the entire cortex for each mouse. The number of EM48-positive aggregates within the indicated regions was normalized to the number of NeuN-positive neurons and included in the analysis. Each data point in the corresponding graphs represents one mouse. For quantification of Gal3 and Cd68 immunofluorescence, five mice per group were analyzed. Coronal sections at matched anatomical levels were imaged, and six systematically selected, non-overlapping fields within the striatum were captured per section. Measurements from these six images were averaged to generate a single value for each mouse. Each data point in the graphs therefore represents one biological replicate.

Neuro-2a cell transfection and drug treatment

Neuro-2a cells were cultured in DMEM supplemented with 10% (v/v) fetal bovine serum (FBS) and 1% penicillin/streptomycin (Gibco), and maintained in a humidified incubator at 37°C with 10% CO_2. Cells were transfected with a mutant huntingtin exon 1 construct containing 109 glutamine repeats fused to GFP (109Q-HTT_{Ex1}-GFP) using T-ProNTR II (JT97-N002M) for 24 hours. Following transfection, cells were treated with vehicle or 10 μM Berbamine hydrochloride for an additional 24 hours. Cells were then fixed, stained with phalloidin (1:400, A12380, Invitrogen) and Hoechst, and subjected to confocal imaging. Image analysis was performed using MetaMorph software. Each data point in the corresponding graphs represents one set of cells. The percentage of cells exhibiting 109Q-HTT_{Ex1} accumulation and the average intensity per cell were quantified specifically in Phalloidin-positive cells.

Western blotting

Western blotting was performed to analyze protein expression levels in brain tissue and cultured cells as detailed previously.[7] Cell samples were lysed with RIPA lysis buffer, while tissue samples were prepared with the AllPrep® DNA/RNA/Protein Mini Kit (80004, Qiagen). Lysates were subjected to SDS-PAGE and transferred onto PVDF membranes. Membranes were incubated overnight at 4°C with primary antibodies, including Gal3 (B2C10[8]), DARPP-32 (1: 1000, #2302,

Cell Signaling), and actin (1: 1000, A2066, Sigma). Following primary antibody incubation, membrane was treated with HRP-conjugated secondary antibodies and visualized using chemiluminescence detection. For Western blot analysis of brain samples, tissue lysates were collected from five mice per group. Due to the number of samples, the 20 lanes were run on two gels. Each gel contained matched samples from all four experimental groups (WT plus vehicle, WT plus Berbamine hydrochloride, R6/2 plus vehicle, R6/2 plus Berbamine hydrochloride) to ensure internal consistency. Each lane represents one biological replicate, corresponding to one mouse. Band intensities were quantified using ImageJ software (NIH). For each sample, the intensity of the target protein band was normalized to the corresponding actin loading control from the same lane. Quantification was performed individually for each mouse. Images of full-length gels are provided in Supplementary Materials.

RNA sequencing and analysis

Striatal brain tissue was collected from treated and control mice for RNA sequencing to assess gene expression changes. Total RNA was extracted using the AllPrep® DNA/RNA/Protein Mini Kit (80004, Qiagen) according to the manufacturer's protocols. The integrity of the RNA samples was evaluated using the Bioanalyzer 2100 (Agilent Technology, USA), and all samples showed an RNA integrity number (RIN) greater than 8.0, which is a threshold for further sequencing. Library construction and sequencing were performed using the in-house-pipeline of Welgene Biotech.

RNA sequencing data were preprocessed to retain genes with a TPM > 1 in at least one sample. To construct the gene co-expression network, an adjacency matrix was first computed using a soft-thresholding power using the pickSoftThreshold function. The adjacency matrix was subsequently transformed into a Topological Overlap Matrix (TOM) to measure the strength of interconnectedness among genes. Genes with high connectivity were grouped together into modules through hierarchical clustering, using the dynamic tree-cutting algorithm (cutreeDynamic), with a minimum module size of 30 genes. Modules eigengenes, defined as the first principal component of each module, that showed a correlation higher than 0.90, were merged to simplify the network and reduce redundancy. The identified 14 modules were assigned unique colors for visualization, and their relationships with the experimental conditions (R6/2 + vehicle, R6/2 + Berbamine hydrochloride, WT + vehicle, and WT + Berbamine hydrochloride) were analyzed through module-trait correlation. To identify hub genes within each co-expression

module, two metrics, Module Membership (MM) and Gene Significance (GS) were applied. Hub genes were defined as those with both high MM (≥ 0.5) and high GS (≥ 0.5), signifying that they are central to the co-expression network of the module and also biologically significant in relation to the conditions. To identify genes co-expressed with Lgals3, TOM values were extracted for Lgals3 from the blue module. Genes with TOM values below 0.8 were considered strongly co-expressed, balancing inclusivity and specificity.

WGCNA was used to identify gene modules with similar expression patterns across the treatment groups and to determine co-expressed gene networks.[9] K-means clustering was performed to categorize the 3,000 most variable genes into distinct clusters based on their expression profiles across treatment groups.[10] Differential gene expression analysis was performed using thresholds of $\log_2$ ratio fold-change greater than or equal to 0.4 or lower than or equal to -0.4 and a *P* value of less than 0.05. Genes with TPM values less than 1 across all experimental groups were omitted from the analysis. Genes meeting these significance thresholds were categorized as either upregulated or downregulated genes for functional enrichment analysis. Functional enrichment analysis was performed using clusterProfiler to explore the biological processes affected by Berbamine hydrochloride treatment.[11] Cell type deconvolution analysis of the DEGs was carried out by Bisque with the murine database by Tabula Muris.[12,13] Unless otherwise indicated, data visualization for the RNA-sequencing analysis was generated using the "ggplot2", "pheatmap" and "EnhancedVolcano" packages in RStudio.

RT-qPCR

Total RNA was reverse transcribed into cDNA by using Superscript III (Invitrogen, USA). qPCR was conducted using SYBR® Green PCR Master Mix (Life Technologies, USA) and was analyzed with either a LightCycler 480 Real-Time PCR System (Roche Life Science) or an ABI QuantStudio 5 Real-Time PCR System (Applied Biosystems). *Gapdh* was used as the reference housekeeping gene to control for quality and quantity. Gene expression levels were normalized to the housekeeping gene and calculated using relative quantification.

Docking

Docking simulations were performed using AutoDock Vina (version 1.5.6).[14] The Gal3 CRD structure was obtained from the Protein Data Bank (PDB ID: 5h9p), while the full-length Gal3

structure was predicted using AlphaFold2. The proteins were prepared by adding Kollman charges and polar hydrogens. Berbamine was docked individually into the protein binding sites using predefined grid boxes. For the CRD, the grid dimensions were set to 72 × 76 × 70 (x, y, z), with the grid center at x = 5.357, y = 0.361, z = -6.261, and a grid spacing of 0.375 Å. For full-length Gal3, the grid dimensions were 126 × 126 × 126 (x, y, z), with the grid center at x = 3.602, y = 2.881, z = -5.048, and the same grid spacing of 0.375 Å. To validate the docking procedure, the known Gal3/TD139 complex was redocked. The results showed that TD139 binds to the S-face of the CRD, with the predicted binding pose closely matching the crystal structure.

Molecular Dynamics Simulations

Molecular dynamics simulations were performed using GROMACS version 2023.3, and the systems were constructed using CHARMM-GUI. The CHARMM36m force field was used for proteins and ligands. The systems were solvated with TIP3P water molecules, and Na^+ and Cl^- ions were added to neutralize the charge and simulate a physiological salt concentration (0.15 M). The simulation box was orthorhombic with a 10 Å buffer from the protein surface. Energy minimization was conducted to remove unfavorable contacts, followed by 100 ps of NVT equilibration at 300 K using a V-rescale thermostat. Subsequently, 100 ps of NPT equilibration was performed at 1 bar using the Parrinello-Rahman barostat. Finally, 10 ns production simulations were carried out with a 2 fs time step, and trajectory data were recorded.

Statistics

Statistical analyses, including Student's t-test, standard deviation, one-way ANOVA, two-way ANOVA, and non-linear regression curve fitting, were conducted using the PRISM 9 program (GraphPad Software, San Diego, CA, USA) and StatPlus 6 (AnalystSoft Inc., VA, USA). A *P*-values of less than 0.05 was considered to be statistically significant. Data are presented as the means ± SEM. No statistical procedures were used to pre-determine the sample sizes.

Study approval

All animal procedures were conducted in compliance with the protocols by the Institutional Animal Care and Utilization Committee of Academia Sinica, Taiwan.

References

1. Lee YH, Lin CH, Hsu PC, et al. Aryl hydrocarbon receptor mediates both proinflammatory and anti-inflammatory effects in lipopolysaccharide-activated microglia. *Glia*. Jul 2015;63(7):1138-54. doi:10.1002/glia.22805
2. Akerblom M, Sachdeva R, Quintino L, et al. Visualization and genetic modification of resident brain microglia using lentiviral vectors regulated by microRNA-9. *Nat Commun*. 2013;4:1770. doi:10.1038/ncomms2801
3. Hsieh TJ, Lin HY, Tu Z, et al. Dual thio-digalactoside-binding modes of human galectins as the structural basis for the design of potent and selective inhibitors. *Sci Rep*. Jul 15 2016;6:29457. doi:10.1038/srep29457
4. Wood NI, Carta V, Milde S, et al. Responses to environmental enrichment differ with sex and genotype in a transgenic mouse model of Huntington's disease. *PLoS One*. Feb 12 2010;5(2):e9077. doi:10.1371/journal.pone.0009077
5. Hsiao HY, Chiu FL, Chen CM, et al. Inhibition of soluble tumor necrosis factor is therapeutic in Huntington's disease. *Hum Mol Genet*. Aug 15 2014;23(16):4328-44. doi:10.1093/hmg/ddu151
6. Siew JJ, Chen HM, Chen HY, et al. Galectin-3 is required for the microglia-mediated brain inflammation in a model of Huntington's disease. *Nat Commun*. Aug 2 2019;10(1):3473. doi:10.1038/s41467-019-11441-0
7. Siew JJ, Chen HM, Chiu FL, et al. Galectin-3 aggravates microglial activation and tau transmission in tauopathy. *J Clin Invest*. Jan 16 2024;134(2)doi:10.1172/JCI165523
8. Liu FT, Hsu DK, Zuberi RI, et al. Modulation of functional properties of galectin-3 by monoclonal antibodies binding to the non-lectin domains. *Biochemistry*. May 14 1996;35(19):6073-9. doi:10.1021/bi952716q
9. Langfelder P, Horvath S. WGCNA: an R package for weighted correlation network analysis. *BMC Bioinformatics*. Dec 29 2008;9:559. doi:10.1186/1471-2105-9-559
10. Ge SX, Son EW, Yao R. iDEP: an integrated web application for differential expression and pathway analysis of RNA-Seq data. *BMC Bioinformatics*. Dec 19 2018;19(1):534. doi:10.1186/s12859-018-2486-6
11. Xu S, Hu E, Cai Y, et al. Using clusterProfiler to characterize multiomics data. *Nat Protoc*. Nov 2024;19(11):3292-3320. doi:10.1038/s41596-024-01020-z
12. Jew B, Alvarez M, Rahmani E, et al. Accurate estimation of cell composition in bulk expression through robust integration of single-cell information. *Nat Commun*. Apr 24 2020;11(1):1971. doi:10.1038/s41467-020-15816-6
13. Tabula Muris C, Overall c, Logistical c, et al. Single-cell transcriptomics of 20 mouse organs creates a Tabula Muris. *Nature*. Oct 2018;562(7727):367-372. doi:10.1038/s41586-018-0590-4
14. Eberhardt J, Santos-Martins D, Tillack AF, Forli S. AutoDock Vina 1.2.0: New Docking Methods, Expanded Force Field, and Python Bindings. *J Chem Inf Model*. Aug 23 2021;61(8):3891-3898. doi:10.1021/acs.jcim.1c00203

Supplementary Figure 1. Details of ultra-high throughput screening for Gal3 inhibition in microglial cells. (Related to Figure 1)

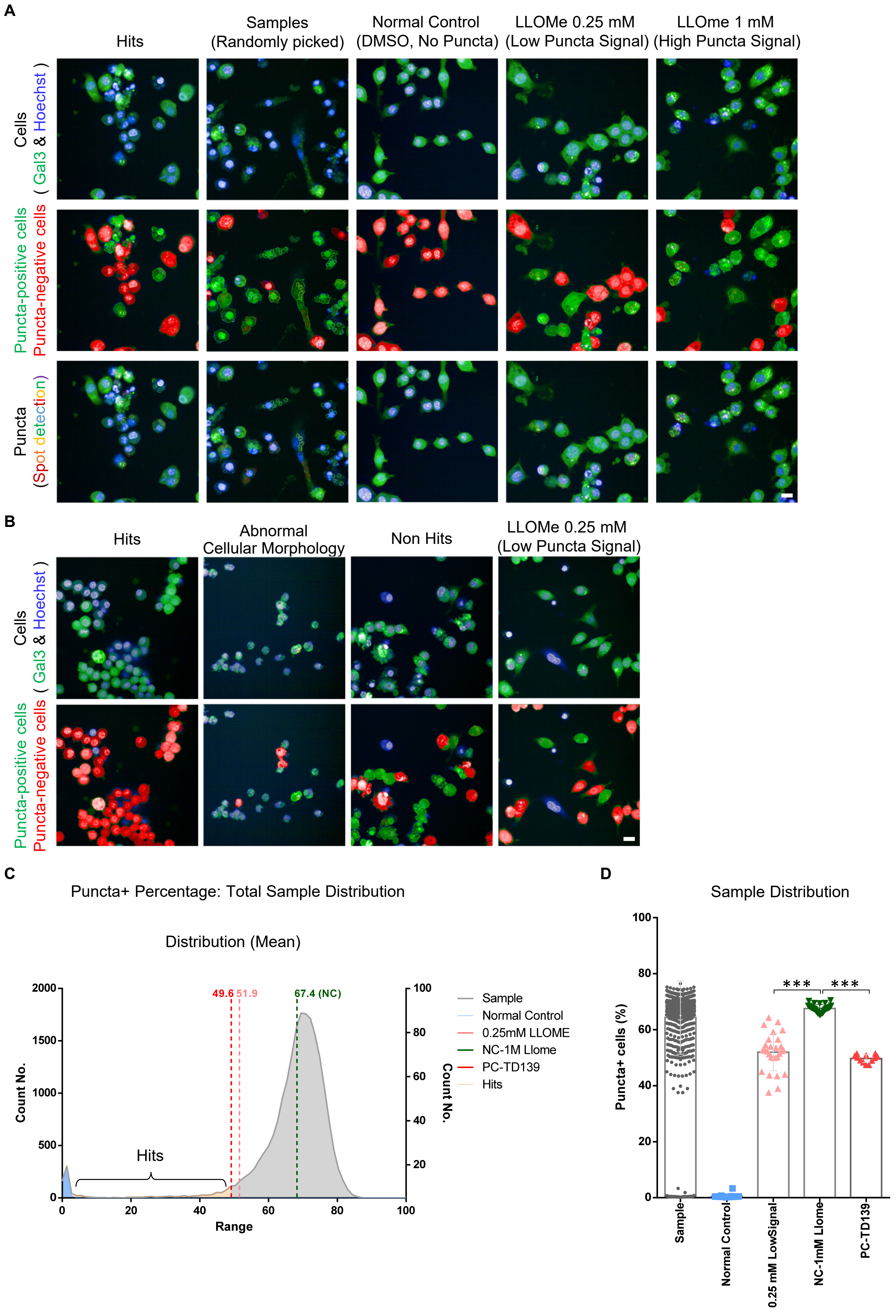

Supplementary Figure 1. Details of ultra-high throughput screening for Gal3 inhibition in microglial cells. (A) Representative images showing Gal3-GFP expressing cells stained with Hoechst (blue), depicting cells with Gal3 puncta (green) and without Gal3 puncta (red) across different sample groups, including hit compounds, randomly picked samples, normal control (DMSO, no puncta), LLOMe 0.25 mM (low puncta signal), and LLOMe 1 mM (high puncta signal). Cells lacking Gal3 puncta after LLOMe treatment are defined as positive screening hits, whereas puncta-positive cells are classified as nonhits. **(B)** Representative images highlighting hits, cells with abnormal morphology, non-hits, and LLOMe 0.25 mM-treated cells, demonstrating the evaluation of cellular morphology and Gal3 puncta formation. Scale bars: 10 μm. **(C)** Distribution of puncta-positive cell percentages across all screened samples, showing the variation between different treatment groups, including normal control, LLOMe (0.25 mM and 1 mM), and positive control (TD139). **(D)** Quantification of puncta-positive cells as a percentage of the total cell population for each group, providing insight into the efficacy of hit compounds. Data were analyzed by unpaired t-test and are presented as mean ± SEM, ***$P < 0.001$.

Supplementary Figure 2. Detection of Z-FA-FMK in the plasma and brain. (Related to Figure 2)

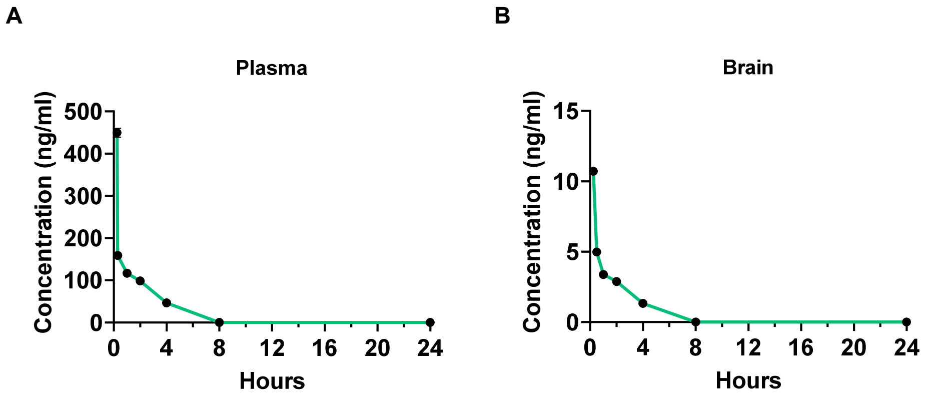

Supplementary Figure 2. Detection of Z-FA-FMK in the plasma and brain. Compounds (5 mg/kg) were injected intraperitoneally, followed by blood and brain collection at specified time points (15 mins, 30 mins, 1, 2, 4, 8, and 24 hours). **(A)** Plasma and **(B)** brain tissue homogenates were analyzed by ultra-performance liquid chromatography/tandem mass spectrometry (UPLC/MS/MS). Each dot represents an individual measurement at a given time point, starting from 15 min.

Supplementary Figure 3. Predicted binding mode of berbamine hydrochloride to galectin-3. (Related to Figure 2)

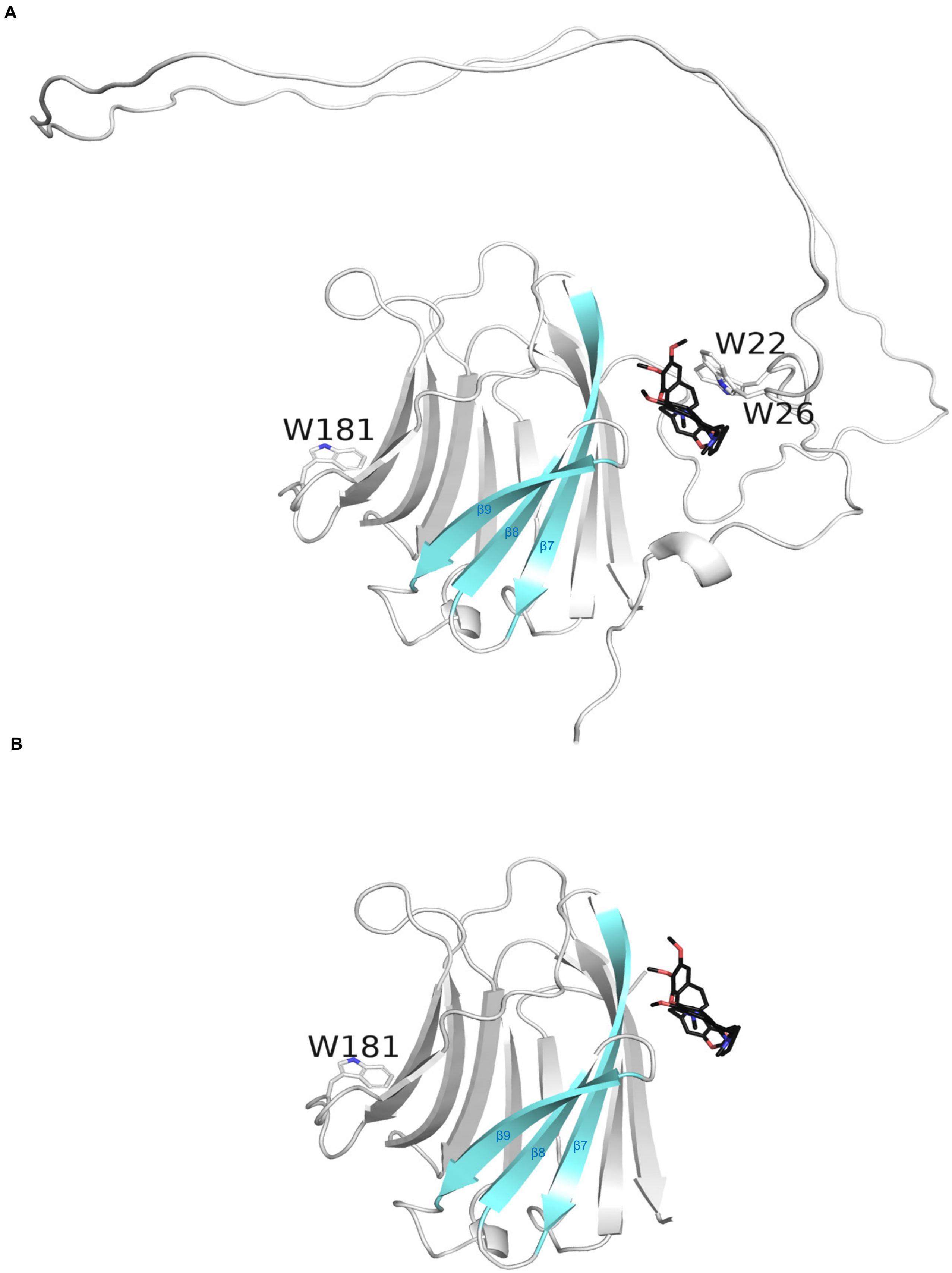

Supplementary Figure 3. Predicted binding mode of berbamine hydrochloride to galectin-3. Docking simulations were performed using AutoDock Vina. Docking models of berbamine bound to **(A)** full-length Gal3 and **(B)** Gal3 CRD are shown. berbamine is predicted to bind to the F-face near β7, β8, and β9, highlighted in cyan. The full-length model includes the NTD, with key residues W22 and W26 may contributing to binding stabilization.

Supplementary Figure 4. Comparison of TD139 binding to galectin-3 CRD and full-length galectin-3. (Related to Figure 2)

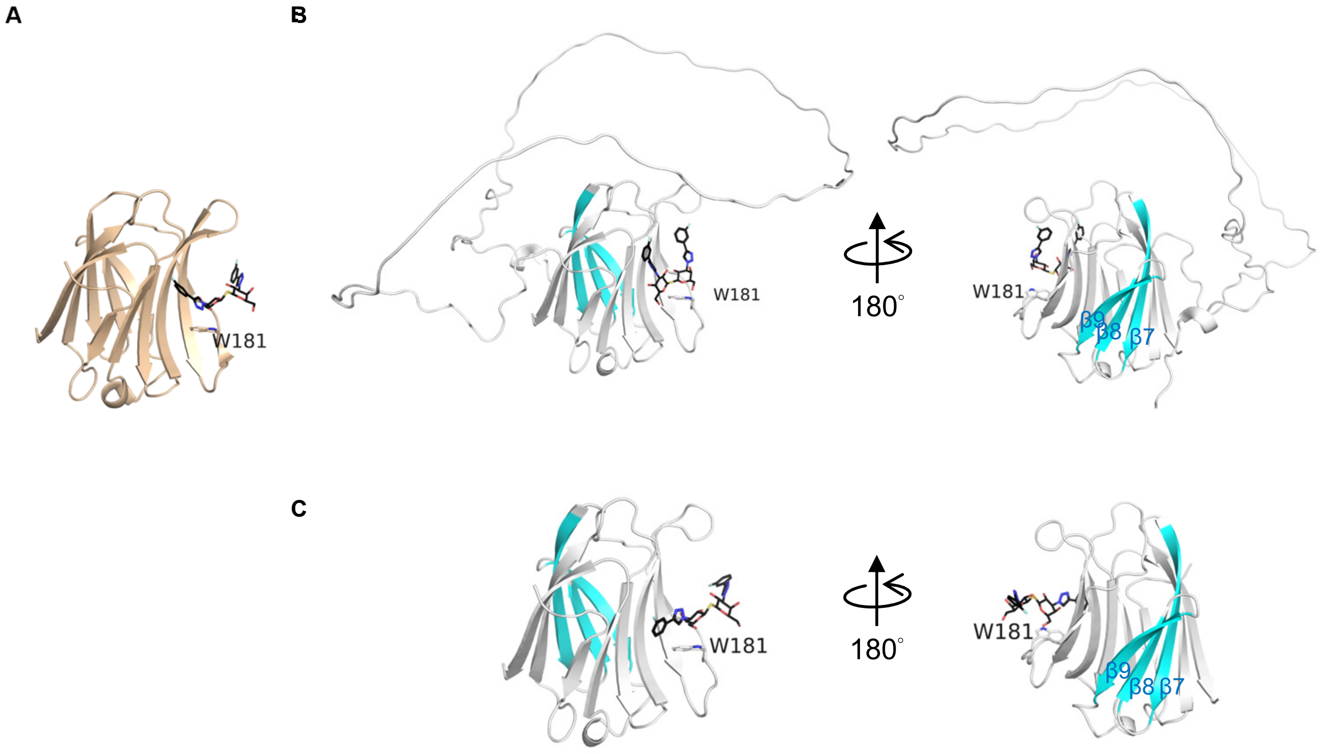

Supplementary Figure 4. Comparison of TD139 binding to Gal3 CRD and full-length Gal3. (A) Crystal structure of Gal3 CRD bound to TD139 (PDB code: 5h9p). **(B-C)** Docking results of TD139 binding to **(B)** the full-length Gal3 and **(C)** the CRD, showing that the compound binds at a position similar to the crystal structure. These results confirm the validity of the docking settings.

Supplementary Figure 5. Effect of berbamine hydrochloride to mutant huntingtin aggregation in Neuro-2a cells and DARPP32 expression in R6/2 mice (Related to Figure 3)

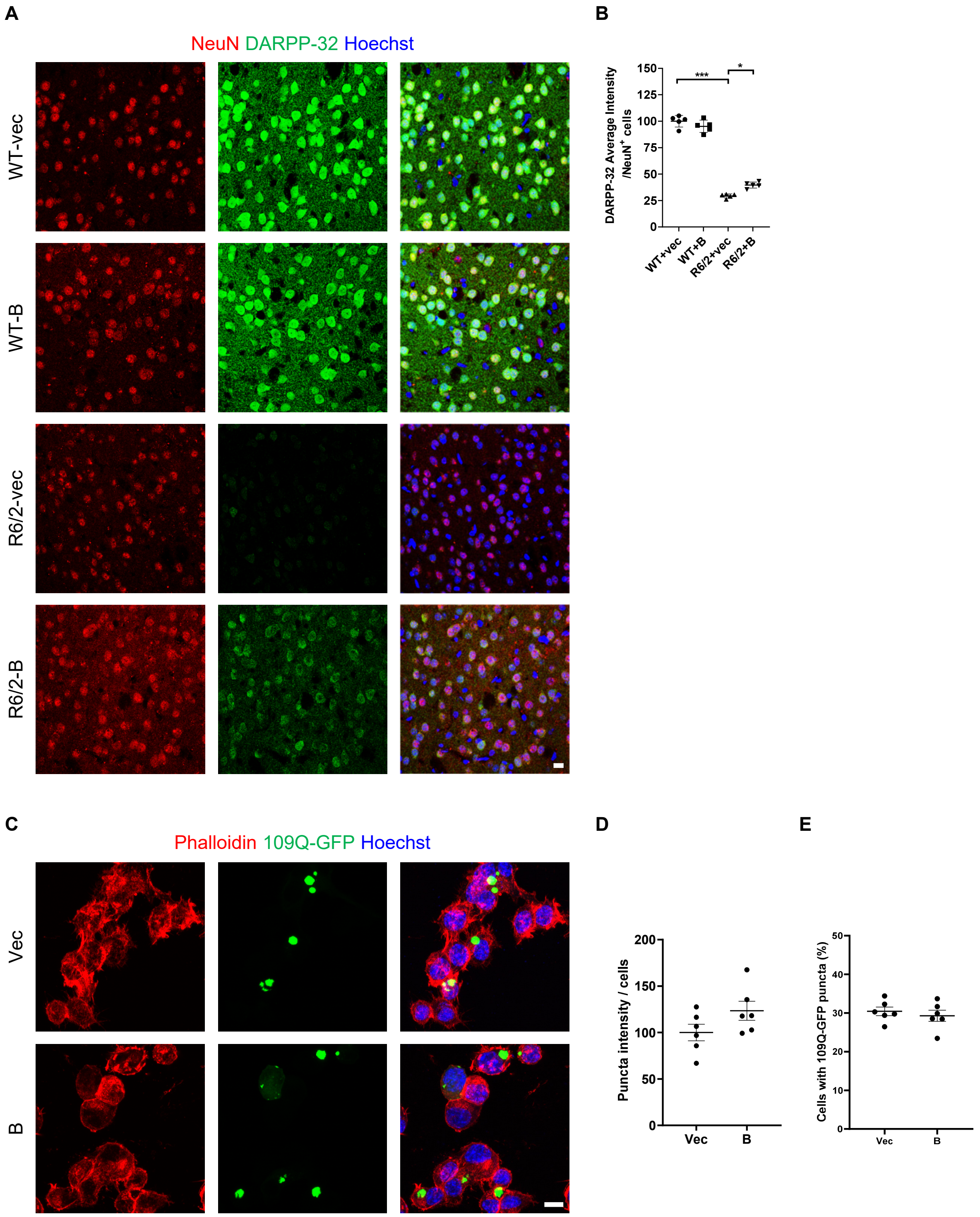

Supplementary Fig. 5. Effect of berbamine hydrochloride to mutant huntingtin aggregation in Neuro2A cells and DARPP32 expression in R6/2 mice. (A) Immunofluorescence staining showing DARPP-32 (green) and NeuN (red) in the striatum. Hoechst staining (blue) was used to label nuclei. **(B)** Quantification of DARPP-32 expression levels in NeuN-positive cells across treatment groups, n = 5. **(C)** Representative confocal images of Neuro-2a cells expressing 109Q-GFP (green), stained with phalloidin to visualize F-actin (red) and Hoechst to label nuclei (blue), treated with vehicle (Vec) or berbamine hydrochloride. **(D-E)** Quantification of 109Q-GFP puncta intensity per cell and the percentage of cells containing 109Q-GFP puncta in Neuro-2a cells treated with vehicle or berbamine hydrochloride, n = 6 independent wells per condition, each data point represents the mean of at least 250 cells. Data were analyzed by unpaired t-test and are presented as mean ± SEM. Scale bars: 10 μm.

Supplementary Figure 6. GO biological processes enrichment of Module brown and yellow. (Related to Figure 4)

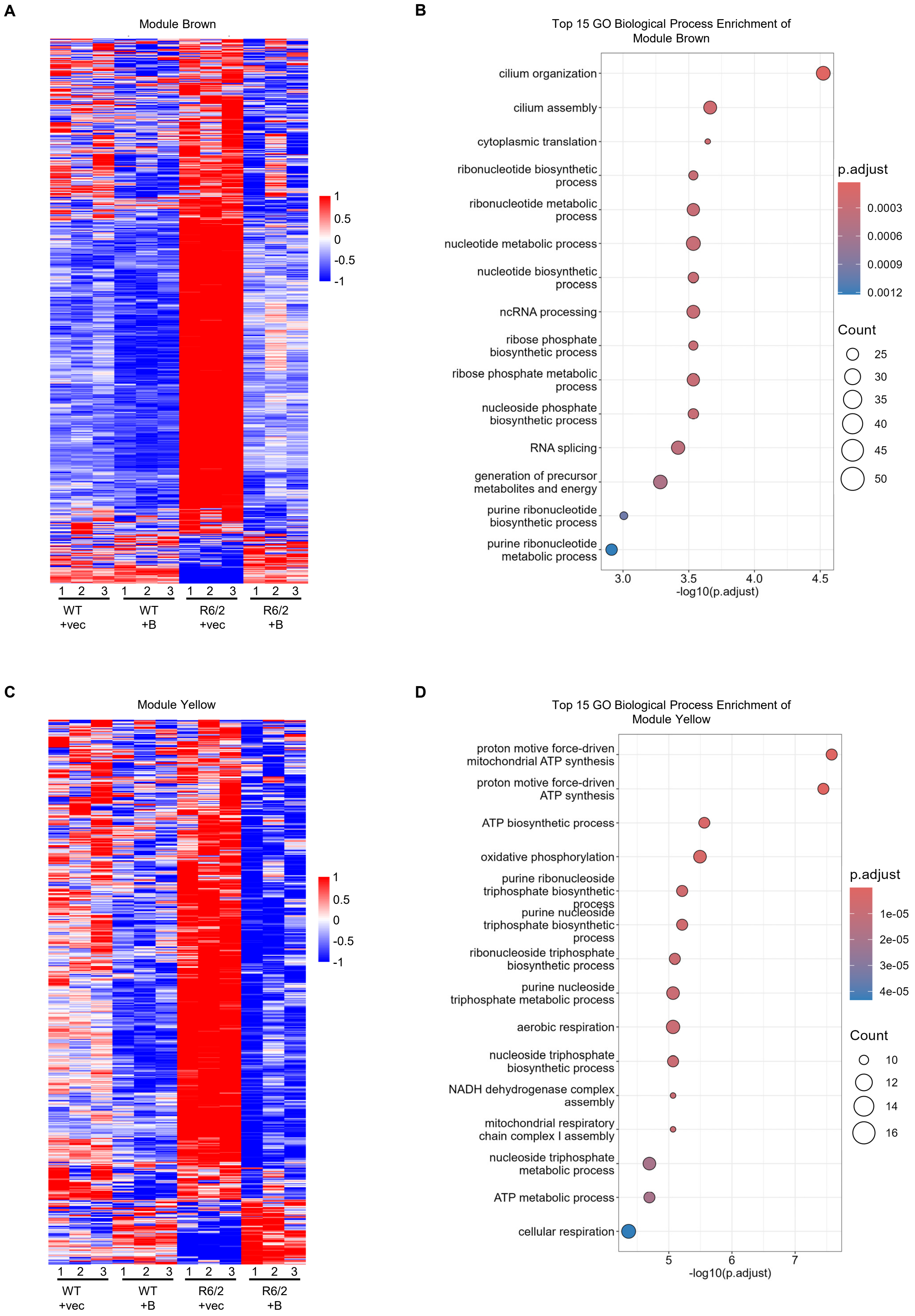

Supplementary Figure 6. GO Biological processes enrichment of Module brown and yellow. Heatmaps and dot plots illustrate gene expressions and top 15 GO biological processes of **(A-B)** Module brown and **(C-D)** Module yellow.

Supplementary Figure 7. K-means clustering and GO analysis of the 3000 most variable genes in the striatum of WT and R6/2 mice treated with berbamine hydrochloride. (Related to Figure 4)

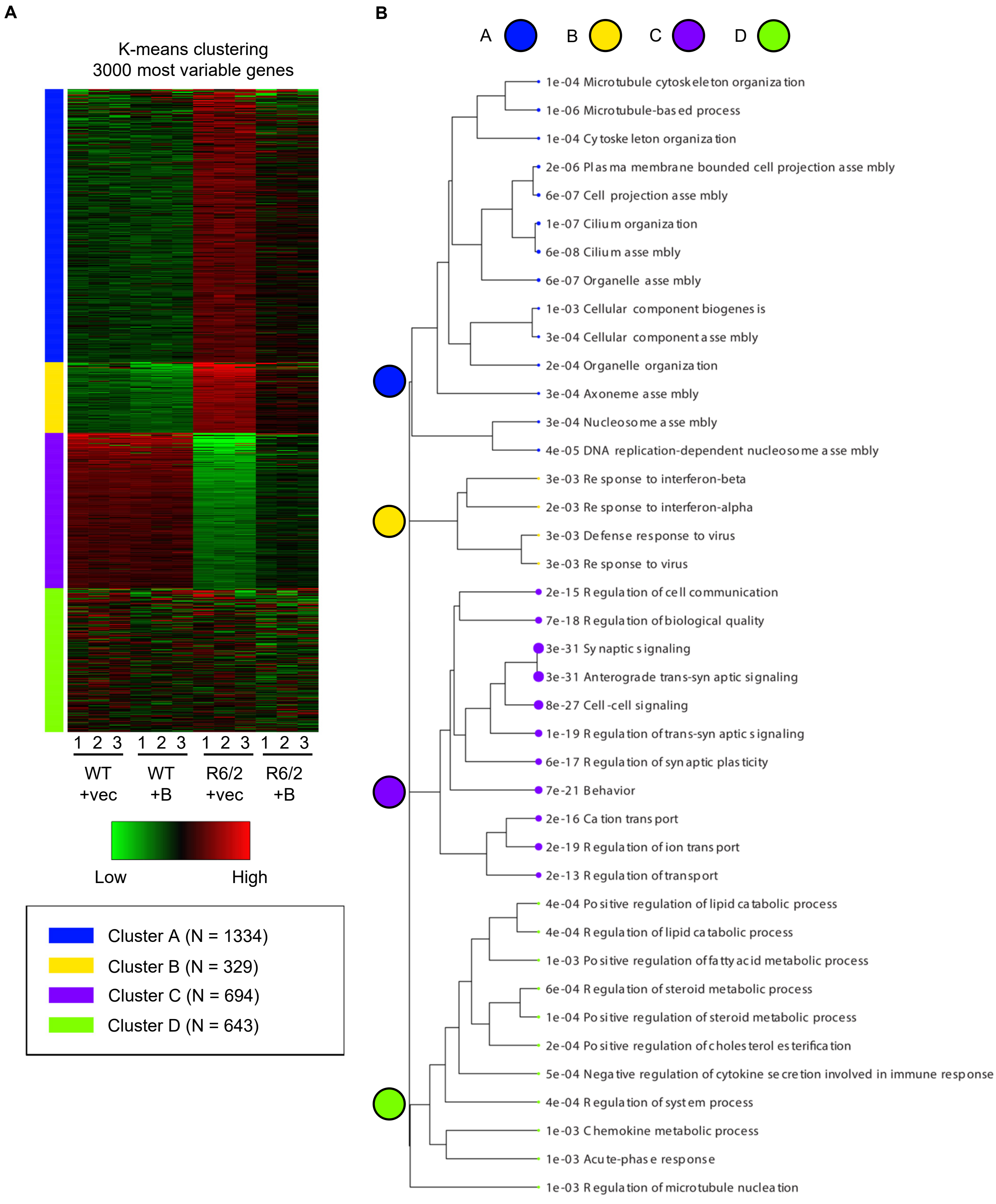

Supplementary Figure 7. K-means clustering and GO analysis of the 3000 most variable genes in the striatum of WT and R6/2 mice treated with berbamine hydrochloride. (A) Heatmap showing the k-means clustering of the expression profiles for the 3000 most variable genes across different treatment groups: WT treated with vehicle (WT+vec), WT treated with berbamine hydrochloride (WT+B), R6/2 treated with vehicle (R6/2+vec), and R6/2 treated with berbamine hydrochloride (R6/2+B). Clustering analysis divided the genes into four clusters (A-D), as indicated by the colored bars. **(B)** GO enrichment analysis of biological processes for genes in cluster A to D. The dendrogram shows enriched biological processes, with cluster identity represented by colored circles: Cluster A (blue), Cluster B (yellow), Cluster C (purple), and Cluster D (green). P-values indicate the significance of enrichment for each biological process.

Supplementary Figure 8. Effect of berbamine hydrochloride on WT mice and cell type deconvolution analysis of DEGs using Bisque. (Related to Figure 5)

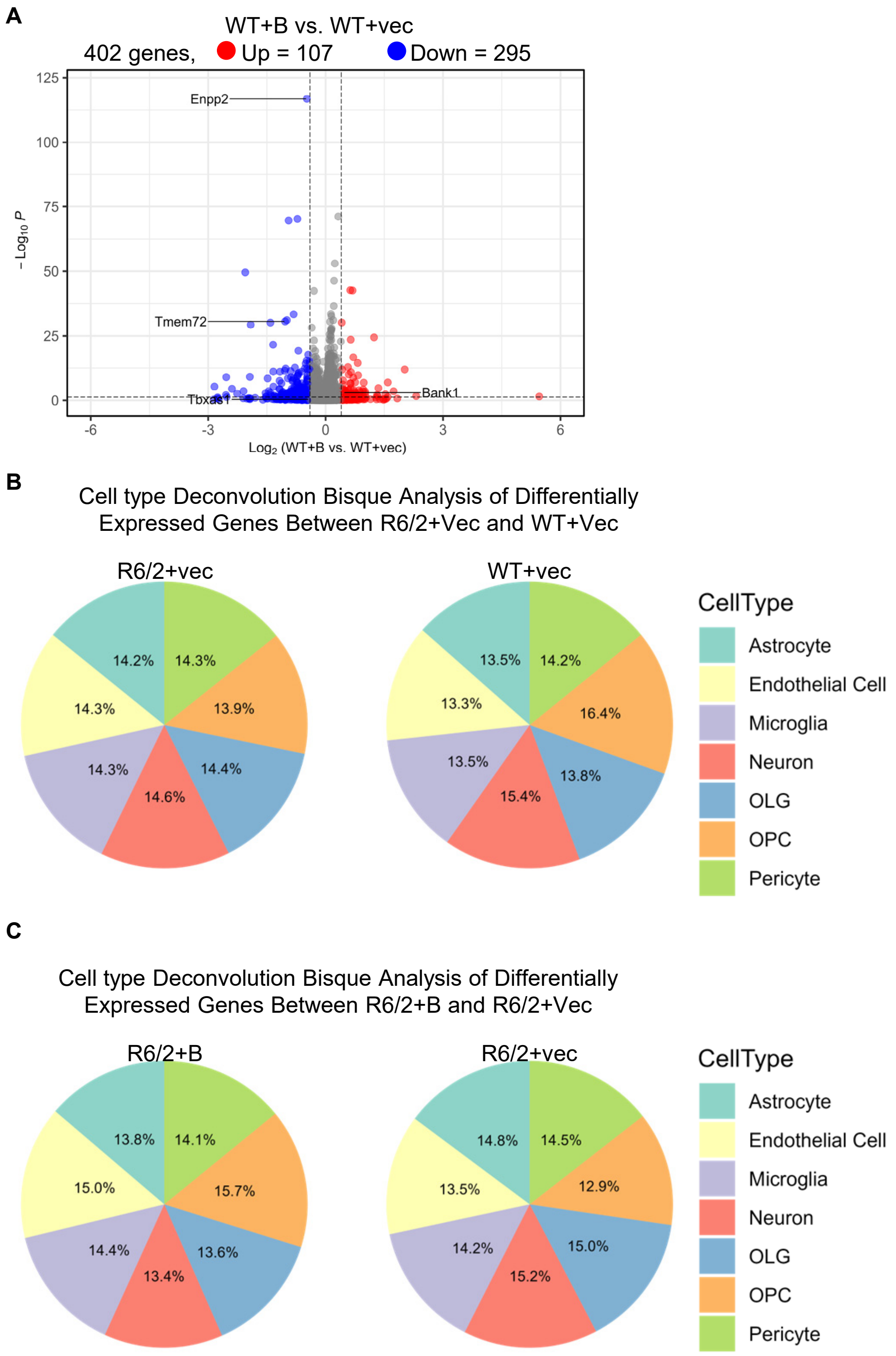

Supplementary Figure 8. Effect of berbamine hydrochloride on WT mice and cell type deconvolution analysis of DEGs using Bisque. (A) Volcano plot showing differentially expressed genes (DEGs) in WT+B versus WT+vec. A total of 402 genes were significantly differentially expressed, with 107 upregulated (red) and 295 downregulated (blue). **(B)** Cell type deconvolution Bisque analysis of DEGs between R6/2+vec and WT+vec. The pie charts represent the estimated proportions of DEGs assigned to different cell types, including astrocytes, endothelial cells, microglia, neurons, oligodendrocytes (OLG), oligodendrocyte precursor cells (OPC), and pericytes. **(C)** Cell type deconvolution Bisque analysis of DEGs between R6/2+B and R6/2+vec. The pie charts represent the estimated proportions of DEGs attributed to each cell type. This analysis provides insight into the cell-type-specific distribution of DEGs and the effects of berbamine hydrochloride treatment.

Supplementary Figure 9. Validation of differentially expressed genes (DEGs) identified by RNA sequencing in striatal brain tissue from WT and R6/2 mice treated with vehicle or berbamine hydrochloride. (Related to Figure 5)

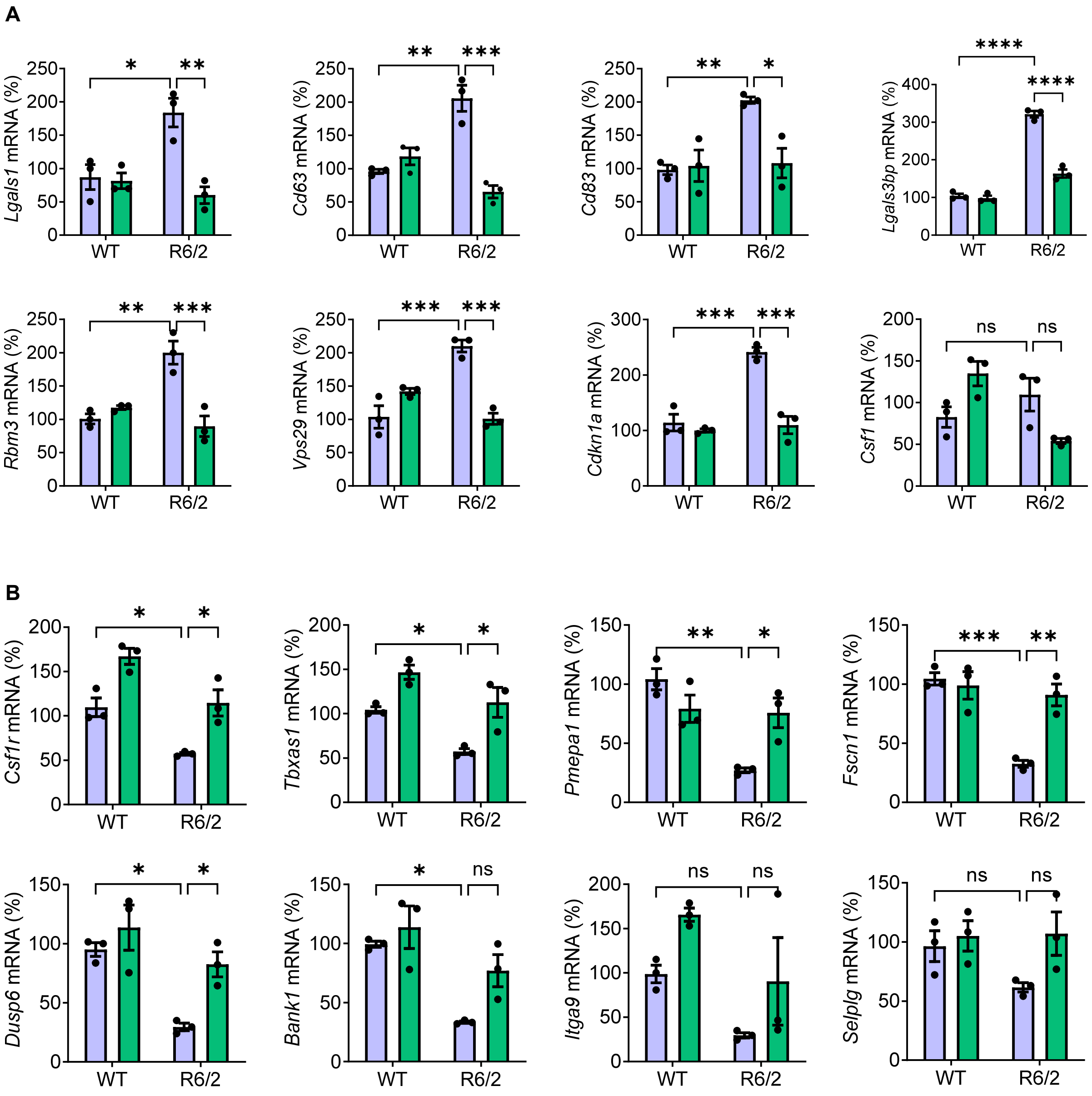

Supplementary Figure 9. Validation of differentially expressed genes (DEGs) identified by RNA sequencing in striatal brain tissue from WT and R6/2 mice treated with vehicle or berbamine hydrochloride. (A) Validation of selected genes that were upregulated in R6/2+vec versus WT+vec and downregulated in R6/2+B versus R6/2+vec, including *Lgals1*, *CD63*, *Cd83*, *Lgals3bp*, *Rbm3*, *Vps29*, *Cdkn1a* and *Csf1* by qPCR, $n = 3$. **(B)** Validation of selected genes that were downregulated in R6/2+vec versus WT+vec and upregulated in R6/2+B versus R6/2+vec, including *Csf1r*, *Tbxas1*, *Pmepa1*, *Fscn1*, *Dusp6*, *Bank1*, *Itga9* and *Selplg* by qPCR, $n = 3$. Data represent mean ± SEM, analyzed by two-way ANOVA with Tukey's test, $*P < 0.05$, $**P < 0.01$, $***P < 0.001$, $****P < 0.0001$, ns = not significant.

Supplementary Figure 10. Effects of berbamine hydrochloride on Gal1 in HD Mice. (Related to Figure 6)

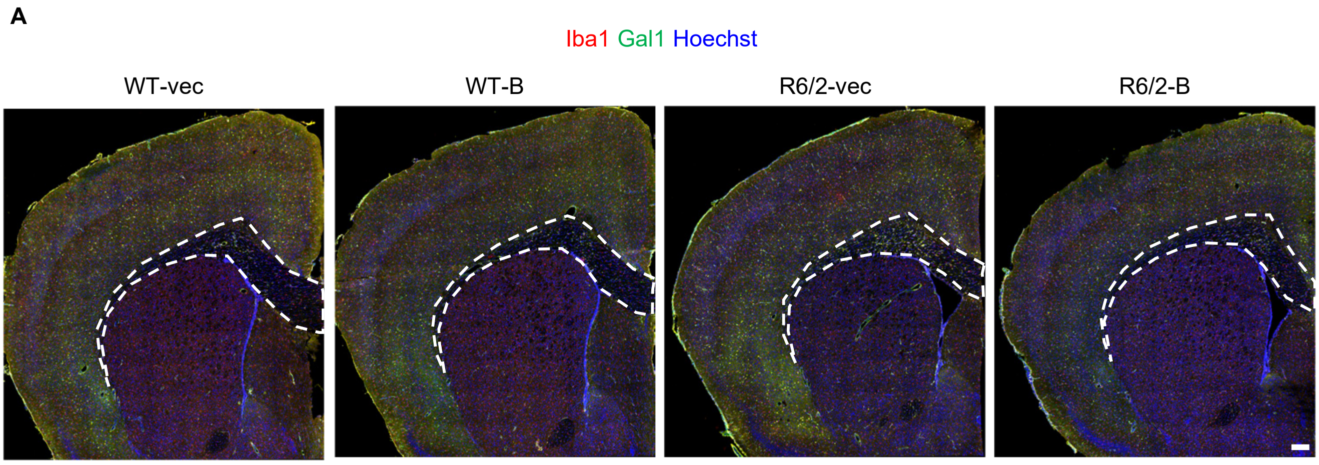

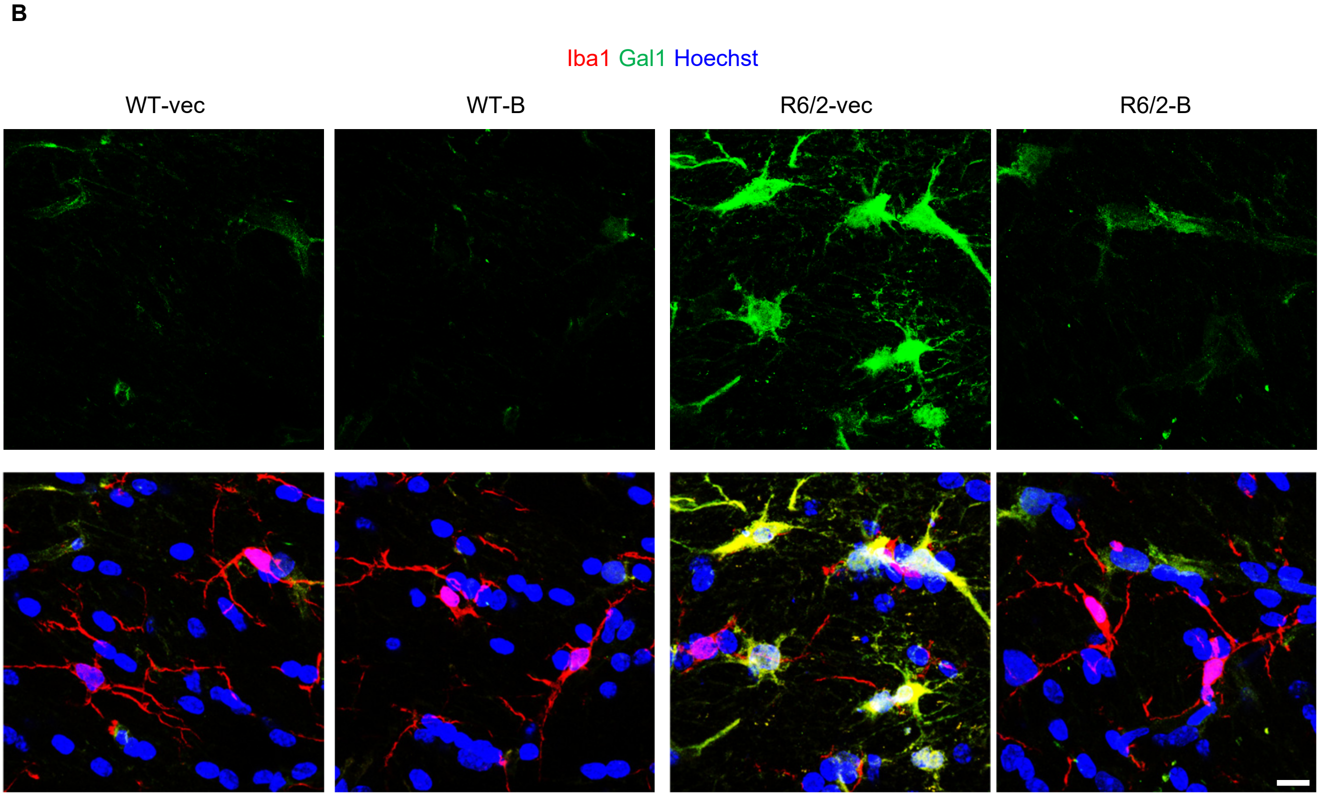

Supplementary Fig. 10. Effects of berbamine hydrochloride on Gal1 in HD mice. (A) Immunofluorescence staining showing Gal1 (green) and Iba1 (red) expression in WT and R6/2 mice treated with vehicle (vec) or berbamine hydrochloride (B). Hoechst staining (blue) was used to label nuclei. Dashed lines delineate the corpus callosum region. Scale bars: 200 µm. **(B)** Higher-magnification images of the corpus callosum regions shown in (A), highlighting Gal1 and Iba1 localization. Scale bars: 10 µm.

Supplementary Figure 11. Full-length gels.

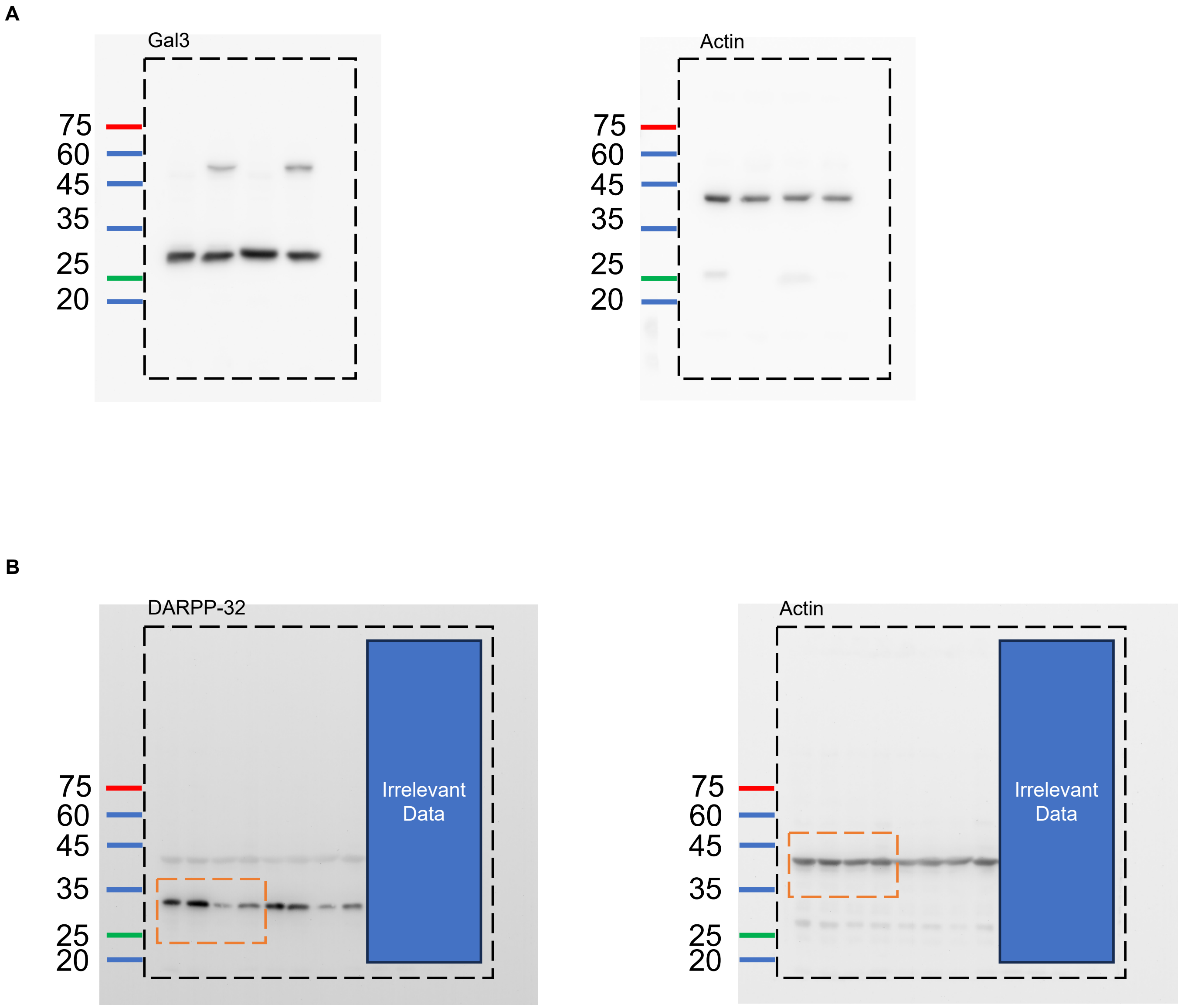

Supplementary Fig. 11 Full-length gels. Full-length gels for Figure 1A **(A)** and Figure 3G **(B)**. Black dashed boxes indicate the boundaries of the membrane, while orange dashed boxes denote the regions presented in the main figures.
